# Rapid Countermeasure Discovery against *Francisella tularensis* Based on a Metabolic Network Reconstruction

**DOI:** 10.1371/journal.pone.0063369

**Published:** 2013-05-21

**Authors:** Sidhartha Chaudhury, Mohamed Diwan M. Abdulhameed, Narender Singh, Gregory J. Tawa, Patrik M. D’haeseleer, Adam T. Zemla, Ali Navid, Carol E. Zhou, Matthew C. Franklin, Jonah Cheung, Michael J. Rudolph, James Love, John F. Graf, David A. Rozak, Jennifer L. Dankmeyer, Kei Amemiya, Simon Daefler, Anders Wallqvist

**Affiliations:** 1 Department of Defense Biotechnology High Performance Computing Software Applications Institute, Telemedicine and Advanced Technology Research Center, U.S. Army Medical Research and Materiel Command, Fort Detrick, Maryland, United States of America; 2 Pathogen Bioinformatics, Lawrence Livermore National Laboratory, Livermore, California, United States of America; 3 New York Structural Biology Center, New York, New York, United States of America; 4 Computational Biology and Biostatistics Laboratory, Diagnostics and Biomedical Technologies, GE Global Research, General Electric Company, Niskayuna, New York, United States of America; 5 Bacteriology Division, U.S. Army Medical Research Institute for Infectious Diseases, Fort Detrick, Maryland, United States of America; 6 Mount Sinai School of Medicine, New York, New York, United States of America; Semmelweis University, Hungary

## Abstract

In the future, we may be faced with the need to provide treatment for an emergent biological threat against which existing vaccines and drugs have limited efficacy or availability. To prepare for this eventuality, our objective was to use a metabolic network-based approach to rapidly identify potential drug targets and prospectively screen and validate novel small-molecule antimicrobials. Our target organism was the fully virulent *Francisella tularensis* subspecies *tularensis* Schu S4 strain, a highly infectious intracellular pathogen that is the causative agent of tularemia and is classified as a category A biological agent by the Centers for Disease Control and Prevention. We proceeded with a staggered computational and experimental workflow that used a strain-specific metabolic network model, homology modeling and X-ray crystallography of protein targets, and ligand- and structure-based drug design. Selected compounds were subsequently filtered based on physiological-based pharmacokinetic modeling, and we selected a final set of 40 compounds for experimental validation of antimicrobial activity. We began screening these compounds in whole bacterial cell-based assays in biosafety level 3 facilities in the 20th week of the study and completed the screens within 12 weeks. Six compounds showed significant growth inhibition of *F. tularensis*, and we determined their respective minimum inhibitory concentrations and mammalian cell cytotoxicities. The most promising compound had a low molecular weight, was non-toxic, and abolished bacterial growth at 13 µM, with putative activity against pantetheine-phosphate adenylyltransferase, an enzyme involved in the biosynthesis of coenzyme A, encoded by gene coaD. The novel antimicrobial compounds identified in this study serve as starting points for lead optimization, animal testing, and drug development against tularemia. Our integrated *in silico*/*in vitro* approach had an overall 15% success rate in terms of active versus tested compounds over an elapsed time period of 32 weeks, from pathogen strain identification to selection and validation of novel antimicrobial compounds.

## Introduction

The threat of emergent, highly infectious, drug-resistant pathogens, whether through genetic engineering of bioterrorism agents or evolution and adaptation of naturally occurring strains, demands capabilities to rapidly screen and identify potential novel antimicrobials for a given pathogen. *Francisella tularensis*, the causative agent of tularemia, is classified by the Centers for Disease Control and Prevention (CDC) as a category A select agent, and its role as a biological weapon is known to have been investigated in the United States, the former Soviet Union, and Japan [1]. Strains of *F. tularensis* [2] are among the most virulent known, requiring only 10 organisms to infect a human intravenously and ∼10–50 organisms through inhalation [3]. Currently, there is no Food and Drug Administration (FDA)-approved small-molecule inhibitor that specifically targets this pathogen, and treatment of tularemia is typically limited to a few antibiotics, such as fluoroquinolones [1]. Although several experimental live-attenuated vaccines [4]–[6] are under development, no vaccine for *F. tularensis* is currently available.

Traditional antibiotic development is characterized by long development times and high costs. For example, a recent review [7] outlining antibacterial discovery efforts at the pharmaceutical company GlaxoSmithKline revealed that 67 high-throughput screening campaigns over the course of six years, at a cost of approximately $1 m a campaign, produced a total of only five lead compounds. Although the time course of a potential outbreak can have tremendous range – from days and weeks in a local outbreak, to months and years in an emerging outbreak abroad – it is clear that traditional approaches are ill-suited to rapidly identify novel drug targets or antimicrobial compounds. There has been extensive work in recent years to identify alternative approaches to drug discovery that leverage bioinformatics and computational biology to identify novel antimicrobials [8]. Here, we demonstrate how a combined computational and experimental approach based on metabolic network modeling, ligand- and structure-based drug design, physiological modeling, and whole bacterial cell-based assays successfully identified novel antimicrobial compounds in a highly abbreviated time period.

Metabolic genome-scale models of bacteria have provided a computational framework for *in silico* simulations to evaluate how metabolic enzymes affect the growth and fitness of an organism under a wide variety of conditions [9]–[12]. The potential for certain metabolic enzymes to be drug targets can be evaluated by simulating the effect of removing the enzyme from the network (*in silico* knockouts) on the organism’s overall metabolism and growth. Furthermore, the effect of a known enzyme inhibitor on metabolism can be explicitly modeled, such as in simulating drug-dose dependent growth inhibition [13]. These modeling frameworks can also be used to identify synergistic effects that arise from combination therapies that inhibit multiple metabolic enzymes simultaneously [14]. Furthermore, the influence of a specific environment on a bacterial organism, such as in a nutrient-rich medium or a nutrient-poor host environment, can be modeled using condition-specific metabolic networks [15]–[18]. The feasibility of antimicrobial compound discovery from analysis of metabolic networks has recently been demonstrated for a range of non-model organisms [19]–[24].

In this study, the target organism was the fully virulent *F. tularensis* subspecies *tularensis* Schu S4 strain, and we implemented a metabolic target identification based on an existing metabolic network reconstructed from the closely related avirulent *F. tularensis* live vaccine strain (LVS) [17]. We applied a systematic multidisciplinary approach to select a small set of enzymes as drug targets, screened ∼20,000 small-molecule compounds to identify putative inhibitors against these targets using ligand- and structure-based drug design, filtered a subset of top-scoring compounds based on desirable pharmacological properties, and experimentally validated a final set of 40 candidate compounds for antimicrobial activity against *F. tularensis* in a whole bacterial cell-based assay. Our discovery pipeline used, by design, a multi-target approach – by targeting multiple enzymes in parallel, we aimed to mitigate the risk that any one enzyme was either unsuitable or ineffective as an antimicrobial drug target. Our combined effort took an aggregated time of 32 weeks and produced six active compounds from a set of 40 tested compounds, with an effective success rate of 15%, which demonstrates the effectiveness of our integrated computational/experimental approach.

## Results

**Figure 1:**
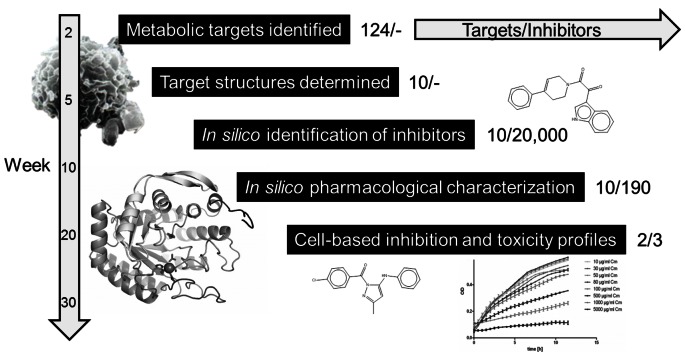
Summary of workflow, timelines, and outcomes. An overview of the major phases of the drug discovery pipelines from target identification, structure determination, *in silico* screening, *in silico* pharmacological characterization and experimental validation. The overall timeline and the number of targets and inhibitors at each phase are shown as well.

We carried out a staggered workflow that combined drug target identification, target selection, *in silico* drug screening, and cell-based experimental validation. Figure 1 shows our overall combined *in silico* and *in vitro* approach, approximate timelines, and results in terms of identified pathogen targets and selected small-molecule inhibitors. First, we constructed a metabolic network model for *F. tularensis* and used it to identify 124 potential metabolic drug targets. Second, we used a target selection scheme to select a subset of 10 drug targets that were predicted to be amenable to small-molecule based inhibition, shown to be essential for growth in other bacteria, and least likely to be associated with adverse effects in humans. Third, we carried out homology modeling and X-ray crystallography to determine the structures of the selected drug targets for subsequent ligand docking. Fourth, we carried out both chemical similarity (ligand-based) and ligand docking-based (structure-based) virtual screening to identify putative small-molecule inhibitors for each target. Fifth, we carried out *in silico* pharmacokinetic (PK) assessment of potential inhibitors identified in screening and selected a set of 40 compounds for purchasing and testing. Finally, we used *in vitro* assays measuring *F. tularensis* growth as well as mammalian cell cytotoxicity to identify non-toxic compounds that showed significant antimicrobial activity.

### Metabolic Target Identification

To create an initial set of candidate drug targets that are likely to affect the growth and viability of the pathogen, we used a genome-scale metabolic model that captures the contribution of each metabolic enzyme toward sustaining biomass accumulation and, hence, growth. In the present analysis, we relied on a genome-scale metabolic model for *F. tularensis* LVS [17], which has recently been augmented and updated with experimental data from *F. tularensis* Schu S4 (K. Amemiya, D. A. Rozak, and S. Daefler, unpublished data). We mapped the LVS metabolic pathways to the Schu S4 strain using existing sequence data and tagged enzymes that abolished accumulation of critical metabolites in the flux-balance model as “essential.” We identified the essential genes as outlined in materials and methods, and the final output of this procedure was the identification of 124 potential drug targets annotated according to the *F. tularensis* subspecies *tularensis* Schu S4 nomenclature. The complete list is provided in Table S1.

### Target Selection

There are certain methodological assumptions inherent in the overall metabolic network analysis that may incorrectly prioritize a given drug target. First, the selection of a subset of genes deemed to be essential for bacterial survival overlooks non-essential genes that may nonetheless be good drug targets. These include virulence factors or other genes that promote infectivity or neutralize protective host immune responses. Second, certain genes that are essential in one environment may be non-essential in another. For example, while most bacteria rely on essential amino acid synthesis in media lacking such metabolites, many are capable of scavenging these amino acids under certain conditions from within the host, thus attenuating the lethality of inhibitors targeting these synthesis pathways. Finally, a reliance on knowledge-based database methods to prioritize drug targets may bias discovery efforts away from novel drugs or drug targets unless one takes care to ensure an adequate mix of novel and known drug targets in the final target selections.

As detailed in materials and methods, we applied bioinformatics analyses to predict whether the enzymes predicted from the metabolic network analysis were druggable. Our assessment of a druggable enzyme was based on the following criteria: *1*) the enzyme is amenable to inhibition by small molecules, *2*) drugs targeting the enzyme will be efficacious and have broad antimicrobial activity, *3*) the enzymes must be structurally characterized so that future structure-based drug design efforts are possible, and *4*) targeting the enzymes with drugs will result in minimal human side effects. Table 1 shows the 31 targets (including the three targets for which we developed the crystal structure in this project) from the initial set of 124 enzyme targets that satisfied at least one of the above criteria to some degree.

**Table 1:** Evaluation for selecting druggable targets.

Gene Locus	Gene Name	Known Chemistries	Essential in Other Organisms	PDB Sequence Similarity	PDB	Human Target*E* Value	Criteria Sum*
FTT0782	fabI	35	10	100	3NRC	–	4
FTT1208	rpiA	16	5	100	3KWM	–	4
FTT1249	nadE	6	3	100	3FIU	–	4
FTT0489c	trxB	9	6	66	1CL0	–	4
FTT0963c	aroG	8	2	–	This work	–	3
FTT0473	accC	13	10	67	2GPW	8×10^−120^	3
FTT0149c	metK	8	10	66	1RG9	3×10^−114^	3
FTT0374c	pyrG	22	10	59	2AD5	2×10^−132^	3
FTT1155c	aroK	2	6	59	1KAG	–	3
FTT0372c	accD	0	10	58	2F9Y	–	3
FTT1161	adk	7	10	56	1AKE	3×10^−57^	3
FTT0581	coaD	8	7	48	1VLH	–	3
FTT1478c	kdsB	5	9	48	3OAM	–	3
FTT0559c	cmk	10	10	47	1CKE	–	3
FTT1468c	nadC	8	1	–	This work	5×10^−35^	2
FTT1681c	lpcA	0	2	–	This work	–	2
FTT1369c	tktA	2	9	99	3KOM	9×10^−43^	2
FTT0942c	folK	0	1	99	3MCN	–	2
FTT1721c	purF	4	3	56	1ECG	7×10^−67^	2
FTT1377	fabF	13	10	55	2GFW	2×10^−84^	2
FTT0387	glmU	4	10	50	2V0L	–	2
FTT0876c	aroC	2	4	47	1UM0	–	2
FTT1154	aroB	2	3	43	3OKF	–	2
FTT0894	purCD	6	5	32	2YS7	6×10^−55^	2
FTT0893	purM	3	1	100	3QTY	3×10^−70^	1
FTT0416	glgA	2	1	53	2R4T	–	1
FTT0789	trpE	0	2	50	1I1Q	–	1
FTT1674	ribH	3	0	45	1ZIS	–	1
FTT1681c	purB	3	2	40	1C3U	–	1
FTT1027c	yrbI	0	0	39	3N1U	–	1
FTT1672	ribB	0	0	34	1KZL	–	1

The gene locus tags correspond to the genes in *Francisella tularensis* subspecies *tularensis* Schu S4. Known Chemistries column refers to the number of inhibitors found in DrugBank [28] and in BRENDA [29] that can be associated with the enzyme. The Database of Essential Genes [54] was used to survey in how many other organisms this gene was annotated as essential for growth. The Protein Data Bank (PDB) [25] column refers to the most closely related protein structure available. We also added in the targets for which no initial homology model existed but, through the course of this work, we developed the corresponding X-ray crystallographic structure. These are referred to as “This work” in the PDB column. The human target *E* value reflects the presence of any human proteins homologous to the bacterial protein, and an absence of any detectable homolog is indicated by “−.” The Criteria Sum column refers to the overall assessment of whether a protein target is suitable for drug development, with the highest number indicating the highest likelihood of being able to develop drugs against the particular target. *Criteria Sum is the number of criteria passed out of four total criteria (see Materials and Methods).

### Protein Structure Determination

For the 31 identified putative targets shown in Table 1, seven targets were already associated with well-resolved and complete, or almost complete, protein structures in the Protein Data Bank (PDB) [25] that we could use directly for structure-based drug design. We constructed and expressed full-length or slightly shortened recombinant proteins for the remaining targets to optimize protein crystallization without removing any functionally important regions. As detailed in materials and methods, three proteins [3-deoxy-d-arabino-heptulosonate-7-phosphate synthase (aroG), nicotinate-nucleotide pyrophosphorylase (nadC), and d-sedoheptulose 7-phosphate isomerase (lpcA)] were readily crystallized, and their structure was determined using molecular replacement. Coordinates were deposited in the PDB as 3TQK, 3TQV, and 3TRJ for aroG, nadC, and lpcA, respectively.

**Figure 2:**
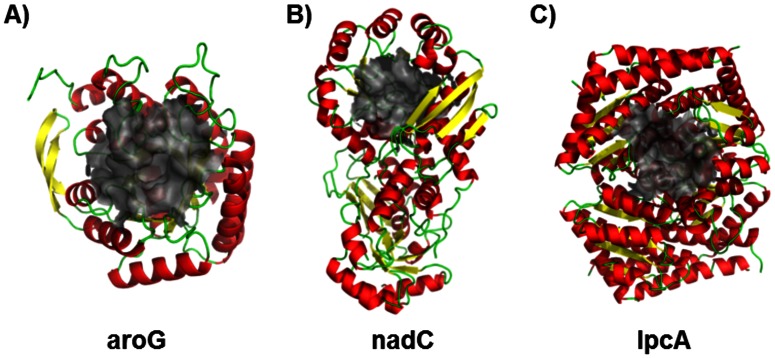
Crystal structures determined in this work. Crystal structures for aroG (A), nadC (B), and lpcA (C) color coded according to their secondary structure elements, with the active site region used in structure-based virtual screening shown as a gray surface.

Figure 2*A* shows aroG (FTT0963c), a phospho-2-dehydro-3-deoxyheptonate aldolase that catalyzes the first step in the chorismate biosynthesis pathway that uses phosphoenolpyruvate (PEP) as one of its substrates. Our version of this protein is a truncated construct [we expressed residues 14–354 (of 370 total residues)], with the missing residues far from the active site and unlikely to influence the function of the enzyme. In the structure, an acetate molecule from the crystallization solution occupies the PEP binding site. The *R* and *R*_free_ values for this structure are 0.189 and 0.227, respectively, at a resolution of 2.3 Å.

Figure 2*B* shows nadC (FTT1468c), a nicotinate-nucleotide pyrophosphorylase. The structure revealed two phosphate molecules bound to the active site. One phosphate molecule is in an identical position to the superpositioned 5′ phosphate moiety from the reaction product analog 5′-phosphoribosyl-1-(β-methylene)pyrophosphate bound to another nicotinate-nucleotide pyrophosphorylase [PDB code 1QPR [26])]. The other phosphate molecule is in close proximity to a second product analog, phthalic acid, found in the same superpositioned enzyme. The *R* and *R*_free_ values for this structure are 0.217 and 0.279, respectively, at a resolution of 2.6 Å.

Figure 2*C* shows lpcA (FTT1681c), a sedoheptulose-7-phosphate isomerase that catalyzes the isomerization of d-sedoheptulose 7-phosphate into d-glycero-d-manno-heptose 7-phosphate. This is the first committed step toward the formation of ADP-heptose, a component of bacterial lipopolysaccharides. This structure of the unliganded state revealed a quaternary arrangement of subunits similar to structures reported in the PDB of other sedoheptulose-7-phosphate isomerases [27]. The *R* and *R*_free_ values for this structure are 0.229 and 0.281, respectively, at a resolution of 2.8 Å. As a critical enzyme in the biosynthesis of lipopolysaccharides, lpcA is of significant interest for current and future antibiotic development.

For the remaining structures for which we were not able to obtain crystal structures in a timely manner, we built homology models, as detailed in materials and methods. All structures were prepared as full atomistic models for use in target evaluation and ligand docking.

### Final Target Selection and *in silico* Small-molecule Screening

Although structural models were available for all 31 targets, we decided against a high-throughput virtual screen strategy against all targets and instead assessed each target based on likelihood of success. The selection was biased toward targets that passed the largest number of criteria for druggability, but was not restricted by it. Given computational and experimental limitations on the number of targets and compounds we could screen, we selected a diverse set of 10 candidate targets across a range of metabolic pathways. Our selected targets, as shown in Table 2, included the three enzymes we were able to crystallize (aroG, nadC, and lpcA), four protein homology model structures [shikimate kinase (aroK), pantetheine-phosphate adenylyltransferase (coaD), chorismate synthase (aroC), and 6,7-dimethyl-8-ribityllumazine synthase (ribH)], and three existing PDB crystal structures [enoyl-(acyl carrier protein) reductase (fabI), NAD synthetase (nadE), and 2-amino-4-hydroxy-6-hydroxymethyldihydropteridine pyrophosphokinase (folK)].

**Table 2:** Target selection and initial compound pools.

Gene Name	EC Number	Protein Name or Function	Structure	Known Chemistries
fabI	1.3.1.9	Enoyl-(acyl carrier protein) reductase (NADH)	3NRC	35
nadE	6.3.1.5	NAD^+^ synthase	3FIU	6
aroG	2.5.1.54	3-Deoxy-7-phosphoheptulonate synthase	Determined 3TQK	8
aroK	2.7.1.71	Shikimate kinase	58% model 1KAG_A	2
coaD	2.7.7.3	Pantetheine-phosphate adenylyltransferase	49% model 1VLJ_B	8
nadC	2.4.2.19	Nicotinate-nucleotide diphosphorylase (carboxylating)	Determined 3TQV	8
lpcA	5.3.1.28	d-Sedoheptulose 7-phosphate isomerase	Determined 3TRJ	0
folK	2.5.1.15; 2.7.6.3	Dihydropteroate synthase	99% model 3MCN_B	0
aroC	4.2.3.5	Chorismate synthase	57% model 1UM0_A	2
ribH	NA	6,7-Dimethyl-8-ribityllumazine synthase	46% model 1VSW_A	3

The metabolic gene targets, their enzyme classification (EC) numbers, and corresponding protein names or functions are shown. The protein structures were derived from three different sources and annotated in the Structure column according to the following criteria *1*) if the structure was previously deposited in the PDB, the corresponding PDB code is given; *2*) if the structure was determined by X-ray crystallography in the course of this work, it is indicated as “determined” and the PDB code is given; or *3*) if it was derived from a homology model the nearest structure’s sequence identity is given as a percentage followed by the primary template used for modeling. The number of known inhibitors we could associate with the protein structure through literature searches is shown in the Known Chemistries column. NA, not available.

To identify potential inhibitory compounds, we *1*) selected compounds in DrugBank [28] and BRENDA [29] databases that were active against that enzyme class, *2*) searched the literature for compound and chemical series that have been tagged as potential inhibitors, and *3*) in the case when no literature support was available, we used the substrate and product to get small-molecule exemplars that had the proper size and atomic groups to interact with the enzyme active site. We then used these compounds to perform a ligand-based search of 57 million compounds that are commercially available from ChemNavigator (www.chemnavigator.com). We identified the 2,000 closest compounds to each set of initial compounds for each target. From these focused screening libraries, we carried out structure-based small-molecule docking using Schrödinger’s Glide software as well as three-dimensional shape-based ligand screening using the OpenEye ROCS and OMEGA programs, for each target, as appropriate (see materials and methods for further details on library construction and screening). We selected top-ranked compounds from both screening approaches for further consideration.

We aimed to identify 20 compounds per target, with equal weight given to docked compounds with good docking poses and compounds that had high shape and chemical similarity. After removing the compounds that we deemed chemically too similar to one another in each target set, we narrowed our search of 20,000 molecules to 190 compounds with putative efficacy against *F. tularensis*.

### *In Silico* PK Assessment

A primary cause of failure in translating promising lead compounds into clinically effective drug candidates is poor PK properties, which lead to insufficient distribution and availability of the compound for treatment. To address this, we implemented a PK parameter filter to make an up-front prediction of the PK properties of each compound and deselect from further consideration compounds whose PK values deviated beyond the typical ranges associated with approved drugs [30]. We used a whole-body physiological-based PK (PBPK) model [31] to determine the volume of distribution (V_d_; in l/kg), clearance (CL; in ml·min^−1^·kg^−1^), mean residence time (MRT; in h), and half-life (*T*_½_; in h) for the compounds that were identified as possible inhibitors. Although quantities calculated by means of PBPK models are macroscopic parameters and are qualitative in nature, they can provide a general indication of whether a compound is likely to pass future pharmacological hurdles.

**Figure 3:**
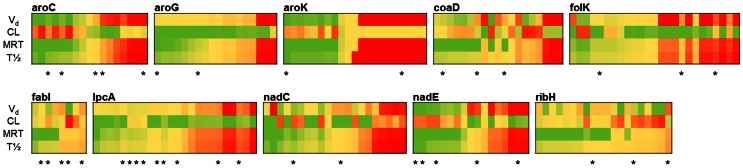
Distribution of calculated pharmacokinetic parameters. Predicted PK parameters for each of the set of 190 candidate compounds are shown. Each column represents one compound, and compounds are grouped according to their target. PK parameter values are represented by a color spectrum ranging from “undesirable” (red) to “desirable” limits (green), defined by the respective 10 and 90 percentile values among the set of Federal Drug Administration-approved drugs for each parameter. For volume of distribution (V_d_), the limits were <0.25 l/kg (red) and >3.30 l/kg (green). For clearance (CL), the limits were >4.5 ml·min^−1^·kg^−1^ (red) and <1.30 ml·min^−1^·kg^−1^ (green). For mean residence time (MRT), the limits were <1.0 h (red) and >14.0 h (green). Finally, for half-life (*T*_½_), the limits were <1.5 h (red) and >13.0 h (green). The 40 compounds selected for experimental testing are highlighted (∗). The putative protein targets are shown in Table 2.

Figure 3 shows the distribution of these parameters across the 10 targets. The parameters are color coded from desirable (green) to undesirable (red) based on the distribution of values among FDA-approved drugs. All 190 selected compounds, their structures, and calculated PK parameters are shown in Table S2. The PK parameters varied considerably among the selected compounds, and we considered a compound eligible if at most one parameter was outside the desirable range. We based our final selection of compounds on computed PK parameters, compound availability, and a price of no more than $10/mg while ensuring that a diverse range of compounds would be tested. The compounds were roughly spread equally among the 10 selected targets, and in the timeframe of the study, we successfully acquired and tested 40 compounds from the larger initial set of 190 selected compounds (Table 3).

**Table 3:** Growth inhibition property of tested compounds.

Target	ID	Compound SMILES Code	Growth Inhibition
aroC	1	[n]2(c3nc(nc(c3nc2)NC4CC4)N)[C@@H]1C[C@@H](C = C1)CO	No
	2	[P]( = O)([O-])([O-])OCN1C( = O)NC(C1 = O)(c3ccccc3)c2ccccc2.O.O	No
	3	[s]1c2c(cc1C(N(O)C( = O)N)C)cccc2	No
	4	FC(F)(F)c1n[n](c(c1)c3ccc(cc3)C)c2ccc(cc2)[S]( = O)( = O)N	No
	5	[n]4(cncc4)c1ccc(cc1)\C = C2\Oc3c(ccc(c3)O)C\2 = O	Yes
aroG	6	NC(Cc1[o]nc(c1)C)C( = O)O	No
	7	[s]1c(ccc1CC(N)C( = O)O)Br	No
	8	O([C@H]1[C@@H](C = CC( = C1)C( = O)O)O)C( = C)C( = O)O	No
aroK	9	Brc1cc(ccc1)N2NC( = O)\C( = C\c3[o]c(cc3)c4c(cccc4)C( = O)O)\C2 = O	No
	10	Fc1ccc(cc1)N2NC( = O)\C( = C\c3[o]c(cc3)c4ccc(cc4)C( = O)N)\C2 = O	No
	11	S1\C( = C\c2cc(c(cc2)OCc3c(cccc3)COc4c(cc(cc4)C = O)OC)OC)\C( = O)NC1 = S	No
coaD	12	[n]21ncc(c2Nc3c(cccc3)C1 = O)C = O	Yes
	13	[n]21nc(c(c2Nc3c(cccc3)C1 = O)C = O)C	Yes
	14	Clc1ccc(cc1)C( = O)[n]2nc(cc2Nc3ccccc3)C	Yes
fabI	15	N2(C( = O)c3c(cccc3)C2 = O)Cc1c(cccc1)N	No
	16	Fc1c(ccc(c1)C#N)Oc2c(cccc2)O	No
	17	Fc1c(ccc(c1)C( = O)N)Oc2c(cccc2)O	No
	18	[n]2(c3c(cc2C)cccc3)CC( = O)NCc1c(nccc1)N(C)C	No
folk	19	N(Cc2nc3c(nc(nc3O)N)nc2)c1ccc(cc1)C( = O)O	No
	20	N(Cc2nc3c(nc(nc3N)N)nc2)c1ccc(cc1)C( = O)[O-]	No
	21	[nH]1cnc(c1C( = O)N)C( = O)N	No
lpcA	22	[N+]( = O)([O-])c1ccc(cc1)NC2OC(C(C(C2O)O)O)CO	No
	23	O1C(C(C(C(C1CO)O)O)O)Oc2cc(c(cc2)C)C	No
	24	Clc1c(ccc(c1)Cl)OC2OC(C(C(C2O)O)O)CO	Yes
	25	N(C2OC(C(C(C2O)O)O)CO)c1ccc(cc1)OCC	No
	26	N(C2OC(C(C(C2O)O)O)CO)c1ccccc1	No
	27	Brc1ccc(cc1)N[C@@H]2OC[C@H]([C@@H]([C@H]2O)O)O	No
	28	[n]2(cnc(c2O)C( = O)N)[C@@H]1O[C@@H]([C@H]([C@H]1O)O)CO	No
	29	N(C2OCC(C(C2O)O)O)c1ccc(cc1)C	No
	30	[n]2(cnc(c2N)C( = O)N)[C@@H]1O[C@@H](C(C1O)O)CO	No
nadC	31	n1c2c(cc(c1C( = O)OCC)C( = O)OCC)C( = O)CCC2	No
	32	NC( = O)COc1c(nccc1)C( = O)O	No
nadE	33	O1[C@@H](CC( = O)c3c1cc(cc3O)O)c2cc(c(cc2)OC)O	No
	34	[n]1(c2nc(nc(c2nc1)O)N)COCCOC( = O)[C@@H](N)C(C)C	No
	35	[N+]( = O)([O-])c1cc(c(cc1)O)\C = N\c2cc(ccc2)O	Yes
	36	Fc1ccc(cc1)NC( = O)C[n]2c3c(c(c2)\C = C\c4nc(nc(c4[N+]( = O)[O-])O)O)cccc3	No
	37	S1[C@H]2N([C@H](C1(C)C)C( = O)[O-])C( = O)[C@H]2NC( = O)COc3ccccc3	No
ribH	38	N2(NC( = CC2(O)c3cc(ccc3)OC)CC)C( = O)c1ccccc1	No
	39	N2(NC( = CC2(O)c3ccccc3)C)C( = O)c1ccncc1	No
	40	N2(NC( = CC2(O)c3ccc(cc3)OC)C)C( = O)c1ccccc1	No

The compounds that were successfully acquired were initially tested for growth inhibition as detailed in materials and methods. The compounds are arranged according to their putative targets (see Table 2), and the SMILES code is given for each compound. Compounds that exhibited growth inhibition in this assay were passed on for an evaluation of their minimum inhibitory concentration (MIC) and cytotoxicity. ID, identifier.

### Growth Kinetic Assays, Minimum Inhibitory Concentrations, and Cytotoxicity

We made an initial qualitative assessment of the ability of each compound to inhibit cellular growth before proceeding with additional quantitative experimental tests. Thus, as detailed in materials and methods, all 40 compounds were evaluated for their ability to affect the growth of *F. tularensis* Schu S4 in Chamberlain’s medium [32] at 37°C. Among the compounds tested in Table 3, six compounds (*compounds 5*, *12*, *13*, *14*, *24*, and *35*) showed activity against the bacteria. The associated growth curves for these compounds are shown in Figure S1. Analysis of the 48-h growth curves showed that the largest difference between the control conditions of no added compound compared with different amounts of added compound occurred at roughly 32 h. The growth at this time point was roughly reduced by at least 40% at the largest amount of compound added (20 µg/ml). Additionally, two of the compounds, *compounds 14* and *35*, completely abolished bacterial growth at this concentration. The other compounds did not affect bacterial growth under these conditions for a range of possible reasons ranging from the lack of efficacy against their putative target, an inability to reach their target, or simply that the target enzyme was not essential under these laboratory conditions.

**Figure 4:**
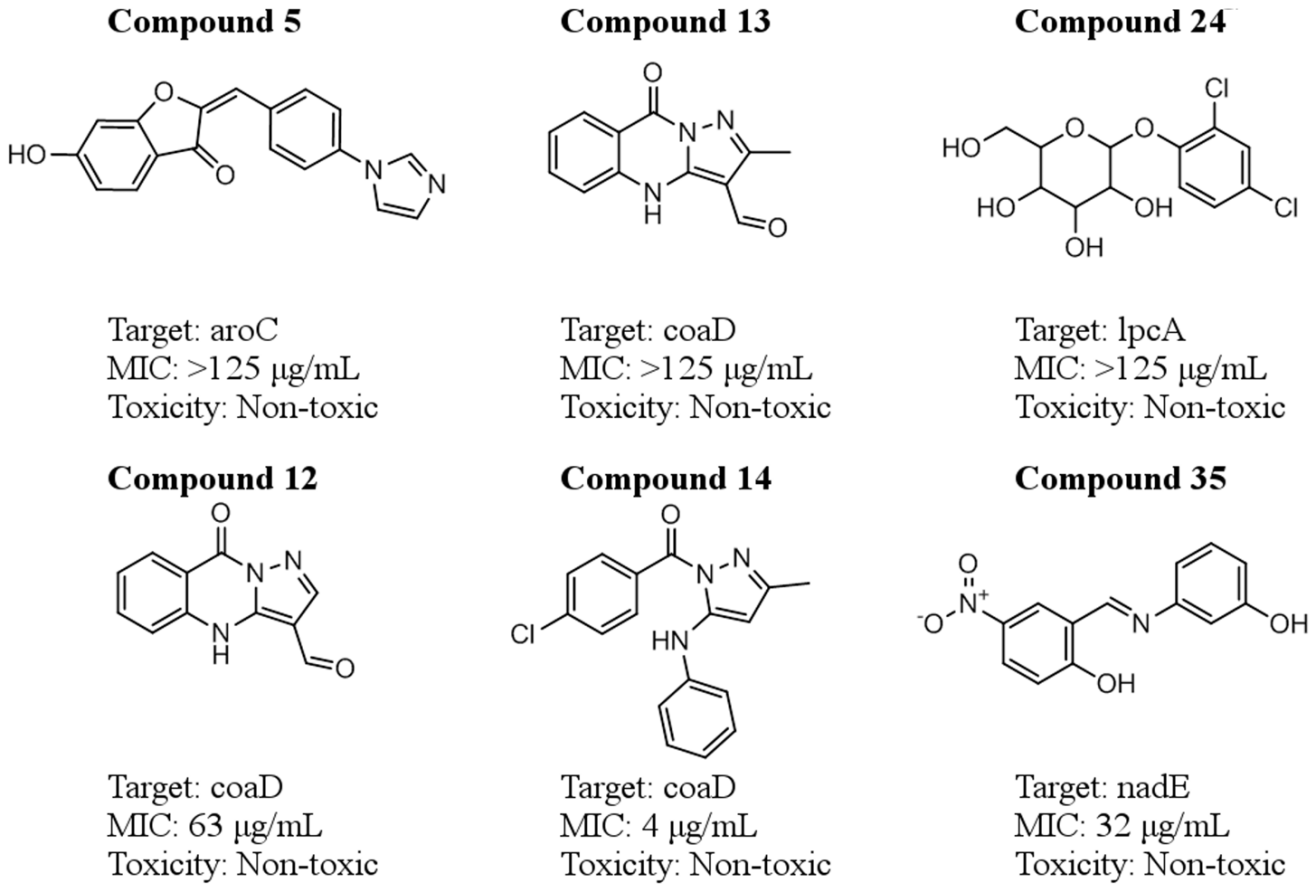
Potency and toxicity of identified antimicrobials against *F.* tularensis. Compound ID, chemical structure, putative target, minimum inhibitory concentration (MIC), and toxicity for each of the active antimicrobials identified in this study.

Based on these results, we evaluated the six compounds for a minimum inhibitory concentration (MIC), i.e., the concentration at which the compound prevents the bacteria from growing, using a preliminary 3-(4,5-dimethylthiazol-2-yl)-2,5-diphenyltetrazolium bromide (MTT) cytotoxicity assay, as described in materials and methods. We used fully virulent *F. tularensis* Schu S4, and all experiments were carried out in biosafety level 3 facilities. Figure 4 shows the chemical structures of each compound and the results of both assays. The computed pharmacological parameters of all compounds (Table S2) were within normally acceptable ranges for future pharmaceutical optimization [30]. *Compounds 14* and *35* showed the lowest MICs, consistent with the growth curve assay results (Table 3).

A preliminary assessment of the compounds revealed a mixed range of potency, toxicity, and novelty with respect to antibacterial scaffolds. Three of the compounds (*compounds 5*, *13*, and *24*) had MIC values >125 µg/ml and could only be considered weakly potent at roughly ∼400 µM. *Compound 35*, a putative nadE inhibitor, was fairly potent but lacked novelty since compounds with similar scaffolds have been discovered to be active against *Bacillus anthracis* [33]. Likewise, *compound 12*, a putative coaD inhibitor, was previously identified in a high-throughput screening study on *Escherichia coli* coaD [34]. However, *compound 14*, also a putative coaD inhibitor, was both a novel compound and the most potent inhibitor identified in the study.

## Discussion

### Aspects of Rapid Drug Discovery

Although “rapid” is typically anathema to good science, the ability to kickstart drug development against novel and genetically modified strains of potential biothreats, such as *F. tularensis*, represents an interesting test case of the scientific community’s ability to quickly produce lead compounds for further development and optimization. In the drug-discovery pipeline presented here, we used *in silico* components that use theoretical and knowledge-based approaches to leverage existing chemo- and bioinformatics information to quickly pare down large screening libraries before time-consuming experimental validation. We found that by simultaneously targeting multiple carefully selected targets we could mitigate the risk of failure of any one target. We also found that enriching and focusing screening libraries with compounds that are chemically similar to those that have been previously identified to inhibit related enzymes was an efficient way of increasing our success rate. Finally, we found that by using *in silico* methods such as homology modeling and enzyme classification prediction, we could expand our list of potential targets beyond those that have been previously crystallized or screened.

The first step in our approach was to identify potential drug targets using a genome-scale metabolic network model of the target *F. tularensis* strain. The identification of essential metabolic enzymes has an accuracy range of 60–90% depending on the organism, level of metabolic network detail, and growth conditions [35]. With this *a priori* target validation accuracy, the likelihood of including a truly essential gene as a target among even a small number of selected enzymes is good. The caveat is that only metabolic enzyme targets are considered. In this study, we adapted an existing metabolic model for a related pathogen, the *F. tularensis* LVS, to *F. tularensis* Schu S4 with minimal effort and generated 124 putative metabolic drug targets within two weeks. Although there is limited data in the literature on essential genes in *F. tularensis*, a systematic study of the related *Francisella novicida* identified 51 of the 124 putative drug targets as essential [36]. Table 4 shows a summary of the time elements, activities, and outcomes of each step in the study.

**Table 4:** Elements and results of the rapid countermeasure discovery project.

*Week*	Project Activities	Targets	Inhibitors
*1–2*	*Francisella tularensis* Schu S4 metabolic enzyme targets	124	–
*3*	Selection of high-likelihood druggable targets	31	–
*4–5*	Protein structure determination/modeling	31	–
*6–10*	*In silico* compound evaluation	10	2,000×10
*11–13*	*In silico* pharmacology evaluation	10	190
*14–29*	Final selection and procurement of compounds	10	40
*18–32*	Cell-based efficacy and toxicity assays	5	6
	Final set of cell-based, low-toxicity, inhibitory compounds	2	3

Target selection based on the existence of known small molecules active against similar enzymes, availability of structures, and lack of human homologs to reduce the potential for unwanted toxicity took another week. The success of generating crystal structures against some of these targets was greatly helped by the availability of genomic DNA, express primer service, and robotics to clone, express, and purify the proteins and to screen crystallization conditions. All 10 enzyme structures derived from new crystal structures, homology modeling, or the PDB were ready for analysis within two weeks of the initial target selection. Genetic studies to validate that these 10 enzyme targets are essential to growth for *F. tularensis* was outside the scope of this study. However, we targeted 10 metabolic enzymes in parallel, to mitigate the risk that any one enzyme is non-essential or unsuitable as an antimicrobial drug target.

The *in silico* selection, analysis, and compound identification of potential small-molecule inhibitors from an initial set of 20,000 compounds to a focused target set of 190 compounds took an additional five weeks. Two additional weeks were spent on an *in silico* PK evaluation of these compounds in a whole-body PBPK model, which allowed us to narrow the compounds to roughly five compounds/target with varying structural diversity and acceptable PK properties.

We received the first set of compounds three weeks after the order was placed and the last set of compounds 8 weeks later, for a total compound acquisition time of 11 weeks for the entire set of 40 selected compounds. The experimental work with the pathogen in biosafety level 3 facilities began as soon as we received the first compounds and was completed 11 weeks later. Compound availability and acquisition was the major bottleneck in our pipeline. We used the online service ChemNavigator, which connects a wide range of compound suppliers and distributors. The ordering, processing, and delivery times, while significantly shorter than they would be if we had individually ordered each compound, proved to be substantial. Furthermore, there was significant attrition with respect to compound availability – approximately 30–40% of requested compounds were either unavailable or extremely expensive. Access to an in-house compound library may significantly decrease compound acquisition time.

In summary, we were able to identify six compounds exhibiting inhibition in whole bacterial cell-based growth assays out of 40 tested compounds against the fully virulent strain within 32 weeks of the start of the project. Three of these compounds (*compounds 12*, *14*, and *35*; Figure 4) had good MIC values and low toxicity and represent good starting points for further lead optimization and animal testing to establish their *in vivo* properties. The project further generated three new crystal structures, one of which [lpcA (FTT1681c)] could be an important novel antibiotic target for future inhibitor development.

### Identified Targets and their Inhibitors

The targets chosen for this study were identified from a metabolic network reconstruction, originally based on the LVS of the pathogen [17]. The identification of essential genes from a network-based analysis of the ability of the pathogen to grow with or without a specific enzyme has limited accuracy based on extensive model system evaluations. However, it was inherent in our analysis that *1*) only the enzymes and reactions included in the model were tested and *2*) adaptive responses to different environmental conditions were not modeled in our approach. We mitigated these risks by considering multiple (ten) targets. This proved important because only inhibitors for roughly half the metabolic targets (four) affected bacterial growth *in vivo*.

The six compounds shown in Figure 4 were selected based on predicted inhibition of nadE, coaD, aroC, and lpcA, enzymes that are important in several distinct metabolic pathways. From a drug development perspective, these enzymatic targets and their associated compounds have different advantages and drawbacks. Importantly, none of these compounds had previously been reported active against any *F. tularensis* species. The following enzymes are putative targets of these lead compounds – *in vitro* validation of inhibition of their enzymatic activity will be a critical next step in their development as candidate antimicrobials. Finally, although there was no indication of resistance to these antimicrobials arising after 48 hours, this is something we can explore more extensively in follow-up work.

#### nadE inhibition

NH_3_-dependent NAD^+^ synthetase (nadE), catalyzes the production of the cofactor NAD^+^, an ubiquitous component of cell metabolism [37]. Reduction of NAD^+^ levels impacts a multitude of biological processes in the cell. *Compound 35*, identified through ligand-based screening, targets this enzyme with an overall MIC of 32 µg/ml and is composed of a phenol group and a 4-nitrophenol group separated by an ethylidenemethanamine group. This compound also has activity against *B. anthracis* Sterne with an enzymatic IC_50_ of 2.5 µM and a MIC of >387 µM [33]. The computed human PK parameters of this compound were in good to moderate ranges compared with other FDA-approved drugs. Furthermore, the compound was found to be non-toxic at the tested 20 µg/ml dose. The potency against *F. tularensis* of this relatively low-molecular-weight compound (249.3 g/mol) renders it a good starting point for further optimization and design.

#### coaD inhibition

Phosphopanethiene phosphatase (coaD) reversibly transfers an adenylyl group from ATP to 4′-phosphopantetheine, yielding dephospho-coenzyme A and pyrophosphate [38]. The reaction is the penultimate step of the biosynthesis of coenzyme A, another cofactor with multiple functional roles in the cell. We found that three compounds, *compounds 12*, *13*, and *14*, all identified through ligand-based screening, had MICs of 63, >125, and 4 µg/ml, respectively. The computed PK parameters were in good to moderate ranges, and all three compounds were non-toxic. These scaffolds are similar to compounds identified by Miller et al. [34] in a high-throughput screen against *E. coli* nadE. Based on potency, PK parameters, and toxicity, these three putative coaD inhibitors represent good starting points for further lead optimization.

#### aroC inhibition

Chorismate synthase (aroC) catalyzes the conversion of 5-*O*-(1-carboxyvinyl)-3-phosphoshikimate to chorismate, an important intermediate in the production of the aromatic amino acids phenylalanine, tyrosine, and tryptophan [39]. *Compound 5*, identified through ligand-based screening, had a MIC of >125 µM and is composed of a hydroxyl-benzofuran and a phenyl-imidazole connected by propene. A similar compound was found to be active against *Streptococcus pneumonia* chorismate synthase inhibitor [40]. Although the computed PK parameters were in the good to moderate ranges and the compound was non-toxic, its relatively high molecular weight and relatively low potency precludes it from being a good lead candidate for further optimization. Furthermore, a drawback of inhibitors of essential amino acid synthesis pathways is that a given pathogen may have mechanisms to acquire these amino acids directly from the host environment.

#### lpcA inhibition

Sedoheptulose 7-phosphate isomerase (lpcA) catalyzes the conversion of sedoheptulose 7-phosphate to d-glycero-α-d-manno-heptose 7-phosphate and d-glycero-β-d-manno-heptose 7-phosphate [27]. Both products are important metabolite intermediates in *F. tularensis* lipopolysaccharide biosynthesis, components integral to assembling a functional outer membrane. *Compound 24*, identified through structure-based ligand docking (a dichlorobenzene joined to a tetrahydropyran via a methoxymethane linker), was presumed to be active against this target, with a MIC of >125 µg/ml. A survey of bioactivities against other pathogens using ChEMBL [41] and PubChem [42] turned up no other recorded activity of this compound. It was non-toxic, but its V_d_ was predicted to be poor. However, given its low molecular weight and ligand efficiency (efficacy vs. molecular weight), further steps could be taken to substitute some of the polar groups, thereby making the molecule more easily accessible to tissues and increasing its V_d_.

### Conclusions

The present work represents an effort to systematically use a genome-scale metabolic network model to rapidly identify druggable targets, perform *in silico* evaluations of targets, and identify small molecules that show efficacy against the pathogen. Although the target list was necessarily limited to the predefined set of enzymes that were included in the network, the network modeling allowed us to identify which of these enzymes were likely critical in sustaining bacterial growth. Metabolic enzymes are among the more traditional antibiotic targets, rendering the organism less competent for growth and more susceptible to attack by host immune defense systems.

Given that the metabolic network was incomplete in describing all possible reactions and adaptations that the organisms may execute, we mitigated the risk of failure by simultaneously evaluating multiple targets. Targets were also assessed upfront for their likelihood of being druggable in terms of existence of known compounds associated with similar enzymes and lack of homology to human proteins to reduce the potential for toxicity. We performed a combined ligand- and structure-based drug discovery effort using a combination of known crystal structures, homology models, and crystal structures developed in the project to identify 190 putative compound/target associations among 10 targets. Selections based partially on computed PK parameters further limited the set we initially assessed for ability to affect *in vitro* pathogen cell growth. The growth inhibition assay weeded out compounds that could not reach their targets, failed to inhibit truly non-essential enzymes, or compounds that just were not efficacious enough. This allowed us to focus our experimental evaluation on just a handful of compounds, i.e., quantitatively evaluate each compound’s potency in a biosafety level 3 facility. Thus, within a time span of roughly 30 weeks we identified six compounds that impacted growth of *F. tularensis* subspecies *tularensis* Schu S4 out of 40 tested compounds, which represented an overall 15% success rate. The identified small-molecule compounds will now form the basis for further optimization and animal work.

## Materials and Methods

### Metabolic Network Modeling and Identification of Essential Genes

We adapted a previously developed network model for *F. tularensis* LVS strain for use for *F. tularensis* Schu S4 by identifying and accounting for genetic differences between the two strains. At the time of this effort, the F. tularensis Schu S4 sequences in GenBank were not fully annotated, and we opted to insert mapped differences between the raw Schu S4 sequences and the LVS metabolic genes into the fully annotated LVS reference genome. The GenBank files for the Schu S4 strain were used to obtain coordinates for genes that contained possible genetic variations compared with the LVS strain. MUMmer 3.18 [43] was used to find single-nucleotide polymorphisms (SNPs), and show-snps [43] was then used to list their coordinates in the genome. A Perl script was used to take each gene containing SNPs and/or indels out of the LVS reference genome and replace the appropriate nucleotides with the ones in the Schu S4 strain. Indels were either deleted or inserted. These new genes were then aligned back to the LVS genome with NUCmer [44] to check that the SNPs were correct. The genes were then translated into amino acid sequences and aligned with the genes in the LVS reference genome using ClustalW [45]. Using this approach, we identified four genes (FTT0196c, FTT1456c, FTT1377, and FTT1373) that showed significant differences between the LVS and Schu S4 strains, and we adjusted the metabolic model accordingly.

Models were analyzed for single lethal (essential) gene knockouts by traditional flux-balance analysis as implemented using the COBRA toolbox [46]. This was achieved by calculating the maximum rates of biomass production for each single gene knockout compared with wild-type strains one gene at a time. The gene and corresponding reaction deletions were implemented using linear programming algorithms following the work of Suthers et al. [47]. The entire computational procedure was carried out in MATLAB to input the stoichiometric matrix, metabolites, reactions, and gene-protein reactions of the model.

### Target Selection Strategy

The four criteria for selecting drug targets among the 124 essential metabolic protein enzymes shown in Table S1 were implemented as follows: for *criterion 1*, we first determined if enzymes were related to other targets for which drugs or other small molecules had successfully been discovered. The sequence of each target was used as a query to run NCBI BLASTP [48] searches of DrugBank [28], [49]–[51] to find related sequences with associated drug molecules. DrugBank protein sequences were deemed significantly related to the query sequence if they had an *E* value of <10^−20^ (a measure of statistical significance) and a hitScore of >99.9 (a measure of alignment quality of query sequence to reference sequence). As an additional step, we assigned an enzyme classification (EC) number to each target sequence by running a BLASTP search of the UniProtKB/Swiss-Prot database [52] to obtain Swiss-Prot gene identifiers associated with each query sequence. We used an *E* value cutoff of 10^−3^ in this case. We then used the Swiss-Prot gene identifiers to search the ENZYME database [53] to obtain EC numbers. Using the EC numbers as queries, we searched BRENDA [29] to obtain inhibitors associated with the EC. We considered *criterion 1* satisfied if five or more inhibitors were found in DrugBank and/or BRENDA.

For *criterion 2*, we carried out BLASTP searches of the query sequence against the Database of Essential Genes (DEG) [54]. We used an *E* value cutoff of 10^−20^ and stored the 10 closest hits. Both the sequence identity of the hits as well as the number of unique hits in the DEG database served as indicators of potential efficacy and broad-spectrum character of drugs used to target the enzyme. We considered *criterion 2* satisfied if at least two organisms in DEG had the gene listed as essential.

For *criterion 3*, we queried each target sequence against a BLASTP database made up of all the protein sequences in the PDB, a database of experimentally determined protein structures [25]. The existence of known structures for similar proteins to a target protein suggests both that the target protein is amenable to experimental structure determination methods and that adequate homologs exist for computational modeling. An *E* value cutoff of 10^−7^ was used, and only the top hit was recorded along with its corresponding protein name, *E* value, and sequence identity (in %) with the query sequence. A hit was considered low, medium, or high quality if it had a sequence identity of <30%, 30–50%, and 50–100%, respectively, to the query sequence. We noted the hitScore and *E* value of the highest quality hit for each target sequence as the criterion for assessing the degree of structural characterization of a given target. We considered *criterion 3* satisfied if a PDB structure with sequence identity of >55% was found.

Drugs designed to inhibit essential proteins in a bacterial pathogen may also inhibit homologous proteins in humans, causing adverse or toxic side effects. To avoid this, we prioritized targets that had no close human homologs. Thus, for *criterion 4*, we queried each target sequence against the NCBI human RefSeq online BLASTP database. In this case, we used an *E* value cutoff of 10^−20^, and we retained the top five hits. We considered *criterion 4* satisfied if no hit was found in the human genome.

All BLASTP searches were written into protocols within Accelrys Pipeline Pilot (accelrys.com/products/pipeline-pilot). Unless otherwise specified, all BLASTP databases were generated locally using FormatDB, part of the NCBI BLASTP software package.

### Protein Structure Determination

#### Protein crystallography

Among a selected subset of 31 protein targets (Table S2), we used X-ray crystallography or homology modeling to characterize the structure of 24 targets that did not have existing structures of the *F. tularensis* protein in the PDB. We first attempted experimental structure determination of all 24 proteins using high-throughput X-ray crystallography. Upon inspection of the target protein sequences, we truncated two sequences to remove unstructured NH_2_- or COOH-terminal regions likely to interfere with crystallization. These truncations affected aroG (FTT0963c), which was shortened from amino acids 1–370 to 14–354, and amidophosphoribosyltransferase [purF (FTT1721c)], which was shortened from amino acids 1–496 to 1–482. We obtained genomic DNA for cloning from BEI Resources (www.beiresources.org) and template DNA from a clone collection held at Arizona State University. We cloned 24 targets by PCR from the genomic DNA and/or clone collection and inserted into three expression vectors designed to produce the target protein with NH_2_-terminal 10 x His, maltose-binding protein, or glutathione-*S*-transferase fusion tags. Given the small number of targets, we skipped small-scale expression trials and moved directly into large-scale expression in *E. coli*; 20 of the 24 targets produced enough protein to prepare crystallization trials within 4 days after we received the primers. Three proteins (aroG, nadC, and lpcA) crystallized rapidly, with initial crystals appearing in 1–2 days. We were able to optimize the crystallization conditions and produce diffraction-quality crystals within days thanks to this rapid crystallization. We collected data sets on all three targets using our in-house X-ray source. Preliminary analysis showed that the three structures could be solved readily by molecular replacement. We used automatic tracing using ARP/wARP [55], and manual rebuilding, and refinement was finished within one week of receiving the primers.

#### Homology modeling

For constructing models of protein that have sequence homology to known structures, we used the Amino acid Sequence to Tertiary Structure (AS2TS) protein modeling program to predict the tertiary structure of the targets shown in Table 1 [56], based on their respective protein sequences. In brief, for each protein sequence, the AS2TS system performs PSIBLAST to search the PDB for templates and produces a set of priority models, each selected based on different criterion (e.g., sequence identity or *E* value). The templates are then analyzed and refined. For example, if amino acids are missing from any of the template structures, Local-Global Alignment (LGA) [57] comparisons and secondary-structure prediction using PSIPRED [58] are used to replace the missing amino acids by grafting suitable structural fragments from identified similar structures or the library of structural motifs. We constructed the backbone structures consisting of the main chain atoms of the proteins using the refined templates. We filled in missing loop regions using structural predictions based on alternate templates. Side-chain atom placements for residues identical to those in the template were directly used in the models, and the remaining side chains were calculated using SCWRL [59]. We extracted functional identifiers (i.e., EC numbers and Gene Ontology terms) from identified PDB template descriptions as annotation information to assist in target selection.

### *In Silico* Identification of Possible Inhibitors

We performed a literature search on each of the enzymes shown in Table 2 to collect small-molecule exemplars for each target. We used DrugBank [28], BRENDA [29], and literature to search for compounds associated with each target and used them to retrieve similar compounds from ChemNavigator (www.chemnavigator.com), a database of 57 million commercially available compounds. We used Pipeline Pilot built-in functional circular substructure fingerprints, termed “FCFP_4,” which are based on circular substructure fragments having a maximum diameter of four bonds, to represent the molecules and calculate Tanimoto coefficients to assess similarity of the ChemNavigator compounds to the query compounds [60]. For folK, we used the knowledge of its substrates and products to select an initial molecular exemplar that could interact with the enzyme, whereas for lpcA, we used the virtual screening result of Umamaheswari et al. [61] against lpcA as a source of exemplar inhibitors. For each target from the results of the ChemNavigator search, we identified the 2,000 closest compounds to our collected set of DrugBank, BRENDA, and literature compounds. For each of these 2,000 compound libraries, we expanded each compound’s conformations and generated a single low-energy three-dimensional structure of each of the exemplars collected using the OMEGA program (www.eyesopen.com) [62], [63]. Using these low-energy structures as queries, we ran the ROCS [64] shape comparison application on each compound library and retained the top 10 hit molecules for each target shown in Table 2. These compounds roughly represent the ligand-based drug design approach. In addition, we used the small-molecule docking program GLIDE [65]–[67] in SP mode to dock the 2,000 compound library to ascertain whether the compounds could favorably interact with the active sites of the enzymes themselves. We retained the top-scoring molecules from GLIDE for each target shown in Table 2. Finally, we combined the most shape-similar compounds and the best binders to retain 190 unique compounds for all targets. These compounds were, thus, predicted to be active against the pathogen either based on complementarity to the binding pockets (GLIDE docking) or based on similarity to compounds known to inhibit related targets (ROCS similarity to small-molecule exemplars).

### PBPK Modeling

We used whole-body PBPK model BioDMET [31] software to calculate PK parameters (Vd, CL, MRT, and *T*_½_) for the molecules that were postulated to have a possible interaction with the selected enzyme targets. BioDMET uses ordinary differential equations to describe how drugs are absorbed, distributed, metabolized, and excreted by the human body at macroscopic and molecular scales. Each major organ in the body is modeled, accounting for the circulation flows of arterial and venous blood and lymph. Physical-chemical properties of a molecule, such as its molecular weight, chemical structure, and charge, affect its permeability and clearance characteristics according to the physics-based equations in the BioDMET model.

For each molecule, we computed the partition [log(P)] and distribution [log(D)] coefficients for pH values ranging from 2 to 11 using the Chemaxon version 5.3 partitioning plugin (www.chemaxon.com). We computed estimates for a molecule’s plasma protein binding and liver microsomal clearance from in-house quantitative structure-activity relationship models and the SMILES string for each molecule. We used these input parameters, along with the molecular weight for each molecule, as input to the BioDMET software (version 3) and performed a standardized simulation run for each molecule. We ran each simulation using a single intravenous injection of 1 g of the molecule administered over 10 s in a 71-kg 40-yr-old man with a body mass index of 24 kg/m^2^. We measured the computed concentration (in mg/ml) of the injected molecule in the plasma at 15-, 30-, 60-, 120-, 180-, 240-, 300-, 360-, 720-, and 1440-min time points. These simulated data were used to compute the values of V_d_, CL, MRT, and *T*_½_ for each molecule.

### *In Vitro* Assays

All experimental work used the fully virulent *F. tularensis* subspecies *tularensis* Schu S4 pathogen (Department of Defense Unified Culture Collection strain FRAN016) in properly accredited biosafety level 3 facilities at United States Army Medical Research Institute of Infectious Diseases.

#### Liquid growth kinetics

We tested each compound at concentrations of 0.31, 0.53, 1.25, 5.00, 10.00, and 20.00 µg/ml. For each concentration, we measured activity against bacterial samples in 30-min increments for 48 h after the addition of the compound. Briefly, scrapings from *F. tularensis* Schu S4 in 25% glycerol stock were streaked onto a chocolate agar plate and incubated at 37°C for 2 days until clearly formed colonies appeared. A single colony from the chocolate agar plate was used to inoculate 3 ml Chamberlain’s medium [32], which was incubated overnight in a 50-ml conical tube at 37°C at 250 rpm. The overnight culture was diluted to 0.1 optical density at 600 nm (OD_600_) in fresh Chamberlain’s medium, and 250-µl aliquots were dispensed onto a 96-well plate arrayed with 5-µl samples of the appropriately diluted compound. Next, 200-µl aliquots of the compound-treated inoculum were transferred to the wells of a Bioscreen C honeycomb plate. Plates were sealed and monitored on the Bioscreen C growth curve machine (Growth Curves USA) for 48 h with moderate continuous shaking and OD_600_ scans every 30 min.

#### MIC

Compounds showing activity in the growth curve experiments were subsequently evaluated in MIC and cytotoxicity assays. In the MIC assay, serial dilutions of the compound were made in Mueller-Hinton II cation-adjusted broth (MHB; Becton Dickenson) growth medium [68]. A modified microdilution method of National Committee for Clinical Laboratory Standards guidelines was followed [*Methods for Dilution of Antimicrobial Susceptibility Tests for Bacteria That Grow Aerobically* (5th ed.) 2000, approved standard M7-A5, National Committee for Clinical Laboratory Standards]. Cultures of *F. tularensis* Schu S4 were grown for 2 days at 37°C on Remel chocolate agar plates (ThermoFisher Scientific). Fresh growth from a plate was used to inoculate MHB containing BBL IsoVitaleX (BD Diagnostics) as recommended by the manufacturer. The optical density of the bacterial suspension was adjusted to 1×10^6^ colony-forming units/ml, and 0.05 ml of the adjusted bacterial suspension was added to each well in triplicate in Nunc 268200 round-bottom 96-well plates (Fisher Scientific). Plates were incubated for 44–48 h at 37°C. After incubation, plates were analyzed for growth at 600 nm and individually visualized to verify growth or lack of growth to identify the end point. MIC was determined as the lowest concentration of the compound that completely inhibited bacterial growth.

#### Cytotoxicity assay

We monitored cytotoxicity using a modified MTT assay [69] and mouse leukemic monocyte macrophage RAW 264.7 cells. This assay measured the reducing potential of the cells using a colorimetric reaction. Reductase enzymes in viable cells will reduce the MTT reagent to a colored formazan product. Into a 96-well flat-bottom plate (Costar no. 3596, Fisher Scientific), 0.2 ml of RAW 264.7 cells at 2.5×10^5^ cells/ml in Dulbecco’s modified Eagle medium (DMEM; Gibco Invitrogen) containing 10% fetal calf serum (FBS), penicillin (100 U/ml), and streptomycin (100 µg/ml) were added, and cells were incubated overnight at 37°C with 5% CO_2_. After an overnight incubation, cells were rinsed twice with PBS, and 0.2 ml of DMEM without FBS and antibiotics were added, and cells incubated for 1 h at 37°C with 5% CO_2_. DMEM was removed, and 0.1 ml of fresh DMEM without FBS and antibiotics but containing dilutions of the test compound were added. Cells were then incubated overnight at 37°C with 5% CO_2_. After an overnight incubation (∼20 h), the medium was removed, 0.1 ml of DMEM with MTT (0.08 ml DMEM plus 0.02 ml MTT at 5 mg/ml) were added to each well, and cells were incubated for 2 h at 37°C plus 5% CO_2_. After incubation, 0.1 ml of a solubilizing solution containing 0.2 g/ml of sodium dodecyl sulfate (Sigma-Aldrich) in 50% dimethylformamide (pH 4.6) was added to dissolve the formazan. The absorbance of the colored solution was measured at 570 nm with a reference at 690 nm. The apoptotic molecule staurosporine [70] was used at 10 µM (Sigma-Aldrich) as a positive control in the MTT assay. Stock solutions were prepared at 10 mM in DMSO and diluted in DMEM and used at a final concentration of 10 µM.

## Supporting Information

Figure S1 **Growth curves for the six active compounds.**
(PDF)

Table S1 **Essential metabolic genes.** The *F. tularensis* subspecies *tularensis* Schu S4 nomenclature was used for the gene association.
(PDF)

Table S2 **PBPK results.** *Column 1* shows the ChemNavigator structure identifier, *column 2* shows the molecular SMILES code, *column 3* shows a picture of the molecule, *column 4* shows the volume of distribution (V_d_; in l/kg), *column 5* shows the clearance (CL; in ml·min^−1^·kg^−1^), *column 6* shows the mean residence time (MRT; in h), *column 7* shows the half-life (*T*_½_; in h), and *column 8* shows the putative targets(s).
(PDF)
