# Supplementary material for: Rapid Countermeasure Discovery against Francisella tularensis Based on a Metabolic Network Reconstruction
Source: PLoS One. 2013 May 21;8(5):e63369. doi: 10.1371/journal.pone.0063369 (PMC3660459; doi:10.1371/journal.pone.0063369)
Supplement: Figure S1 — Growth curves for the six active compounds. (PDF) [file pone.0063369.s001.pdf]

Supplemental Figure F1: Growth curves for the six active compounds.

The compounds that were active in the *Francisella tularensis* subspecies *tularensis* Schu S4 growth assay, as described in the MATERIALS AND METHODS, are show at the *left* with the corresponding growth curves at the *right*. The compound identifiers (IDs) are cross-referenced to Table 3, and the corresponding SMILES code is listed under each compound. OD_{600}, optical density at 600 nm.

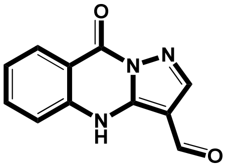

C=O

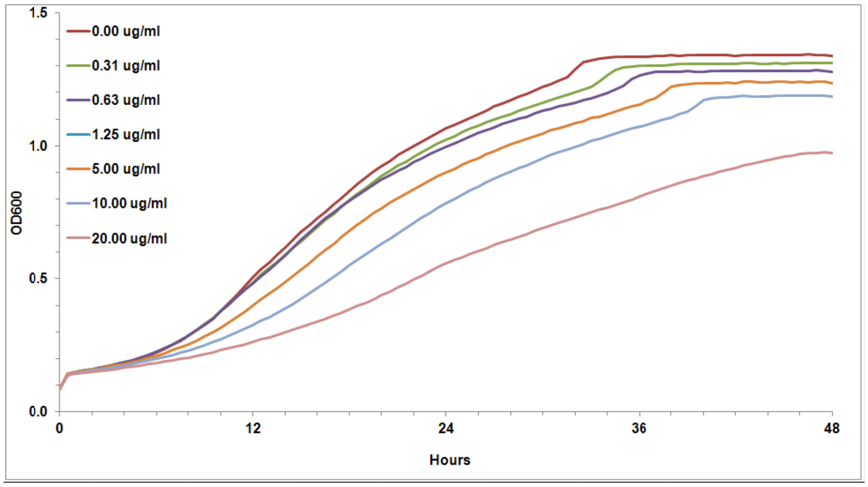

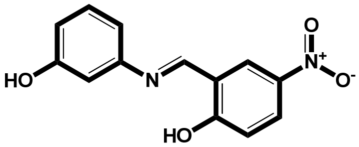

(ccc2)O

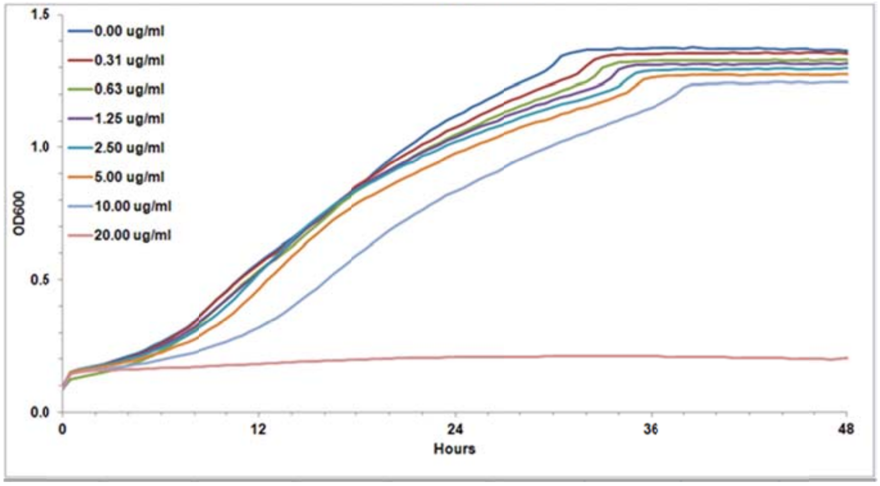

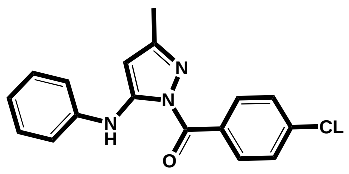

Clc1ccc(cc1)C(=O)[n]2nc(cc2Nc3ccccc3)C

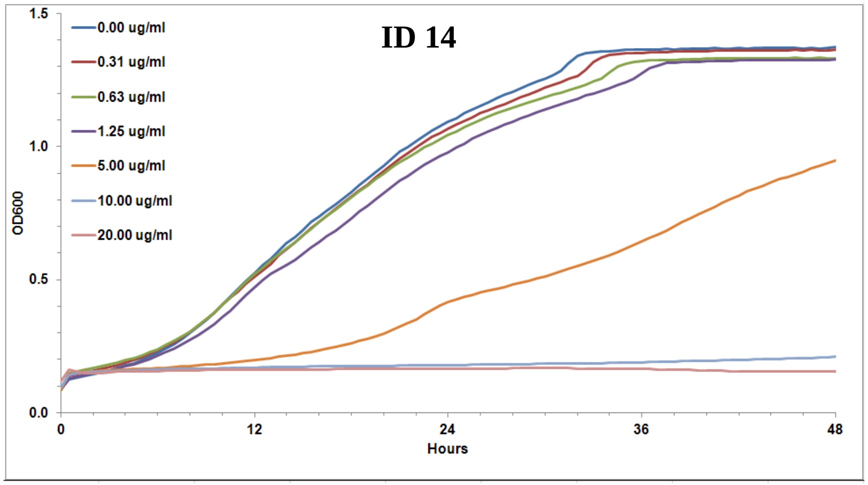

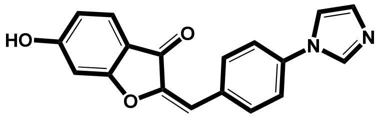

[n]4(cncc4)c1ccc(cc1)\C=C2\Oc3c(ccc(c3)O)C\2=O

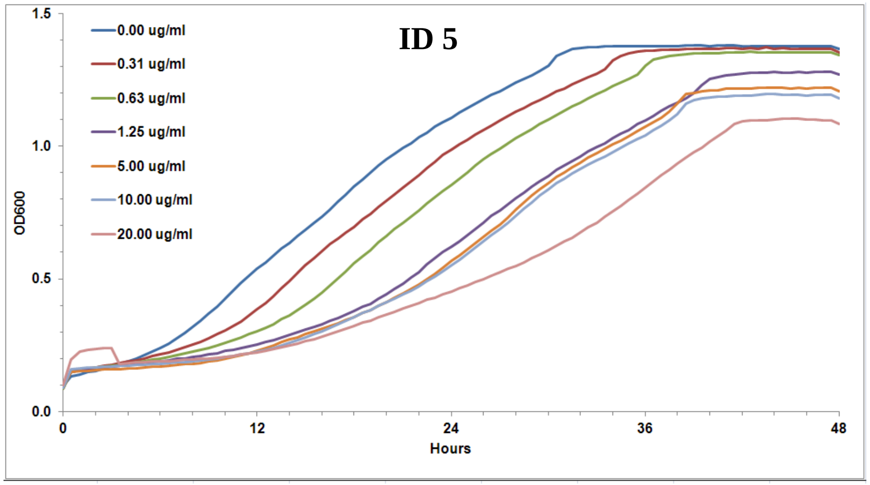

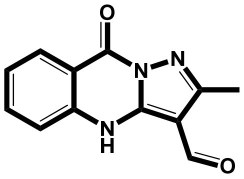

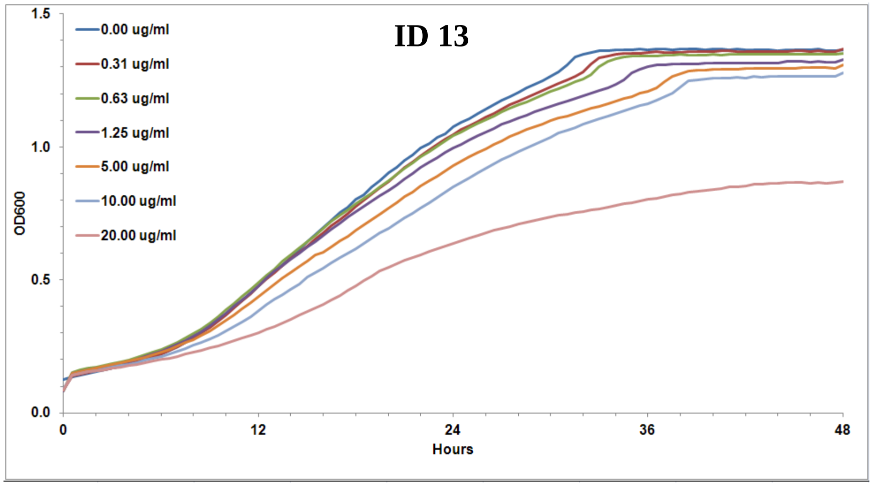

[n]21nc(c(c2Nc3c(cccc3)C1=O)C=O)C

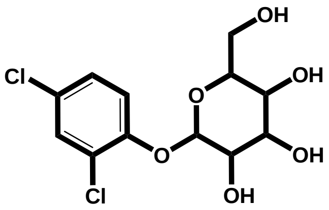

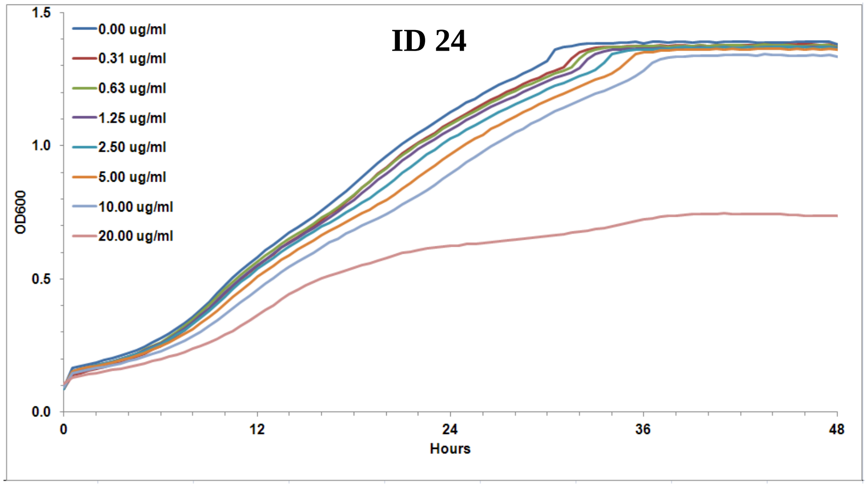

Clc1c(ccc(c1)Cl)OC2OC(C(C(C2O)O)O)CO
