# Supplementary material for: Rapid Countermeasure Discovery against Francisella tularensis Based on a Metabolic Network Reconstruction
Source: PLoS One. 2013 May 21;8(5):e63369. doi: 10.1371/journal.pone.0063369 (PMC3660459; doi:10.1371/journal.pone.0063369)
Supplement: Table S1 — Essential metabolic genes. The F. tularensis subspecies tularensis Schu S4 nomenclature was used for the gene association. (PDF) [file pone.0063369.s002.pdf]

Supplemental Table T1: Essential metabolic genes.

The complete list of all essential metabolic genes generated using the metabolic network model are shown using *Francisella tularensis* subspecies *tularensis* Schu S4 nomenclature. Gene names, Enzyme Classification (EC) numbers, and metabolic pathways are shown when known.

Locus Name	Gene Name	EC Number	Pathway
FTT0149c	metK	2.5.1.6	Cysteine and methionine
FTT0196c	glnA	6.3.1.2	Alanine, aspartate and glutamate, Arginine and proline, Nitrogen
FTT0397	mtn	3.2.2.9	Cysteine and methionine
FTT0411c	aroE2	1.1.1.25	Phenylalanine, tyrosine and tryptophan
FTT0430	speH	4.1.1.50	Cysteine and methionine, Arginine and proline
FTT0471	aroD	4.2.1.10	Phenylalanine, tyrosine and tryptophan
FTT0575	pheA	4.2.1.51	Phenylalanine, tyrosine and tryptophan biosynthesis
FTT0588	aroA	2.5.1.19	Phenylalanine, tyrosine and tryptophan biosynthesis
FTT0834	aroQ	5.4.99.5	Phenylalanine, tyrosine and tryptophan
FTT0876c	aroC	4.2.3.5	Phenylalanine, tyrosine and tryptophan biosynthesis
FTT0946	trpG	4.1.3.27	Phenylalanine, tyrosine and tryptophan biosynthesis
FTT0963c	aroG	2.5.1.54	Phenylalanine, tyrosine and tryptophan biosynthesis
FTT1154c	aroB	4.2.3.4	Phenylalanine, tyrosine and tryptophan biosynthesis
FTT1155c	aroK	2.7.1.71	Phenylalanine, tyrosine and tryptophan biosynthesis
FTT1650c		5.4.99.5	Phenylalanine, tyrosine and tryptophan biosynthesis
FTT1795c	trpC	5.3.1.24	Phenylalanine, tyrosine and tryptophan
FTT1802c	trpE	4.1.3.27	Phenylalanine, tyrosine and tryptophan
FTT0425c	asd	1.2.1.11	Glycine, serine and threonine, Cysteine and methionine, Lysine
FTT0079	glmM	5.4.2.2	Amino sugar and nucleotide sugar
FTT0387	glmU	2.3.1.57	Amino sugar and nucleotide sugar
FTT0416	glgA	2.4.1.21	Starch and sucrose
FTT0789	rpe	5.1.3.1	Pentose phosphate, Pentose and glucuronate interconversions
FTT1093c	talA	2.2.1.2	Pentose phosphate
FTT1208	rpiA	5.3.1.6	Pentose phosphate
FTT1369c	tktA	2.2.1.1	Pentose phosphate
FTT0312c	folA	1.5.1.3	Folate biosynthesis
FTT0371c	folC	6.3.2.17	Folate biosynthesis
FTT0581	coaD	2.7.7.3	Pantothenate and CoA biosynthesis
FTT0916c	ribF	2.7.1.26	Riboflavin
FTT0942c	folK	2.5.1.15	Folate
FTT0943c	folB	4.1.2.25	Folate
FTT0945		4.1.3.-	Folate
FTT0951c	folE	3.5.4.16	Folate
FTT1147c	dfp	6.3.2.5	Pantothenate and CoA biosynthesis
FTT1389	panB	2.1.2.11	Pantothenate and CoA biosynthesis
FTT1432c	ppnK	2.7.1.23	Nicotinate and nicotinamide
FTT1468c	nadC	2.4.2.19	Nicotinate and nicotinamide
FTT1469c	nadA		Nicotinate and nicotinamide
FTT1487	coaE	2.7.1.24	Pantothenate and CoA biosynthesis
FTT1671	ribD	3.5.4.26	Riboflavin
FTT1672	ribB	2.5.1.9	Riboflavin
FTT1673	ribA	3.5.4.25	Riboflavin
FTT1674	ribH	2.5.1.9	Riboflavin

Locus Name	Gene Symbol	EC Number	Pathway
FTT0592	cynT	4.2.1.1	Nitrogen
FTT1028c	ppa	3.6.1.1	Oxidative phosphorylation
FTT0110	lpxK	2.7.1.130	Lipopolysaccharide
FTT0189	lpxC	3.5.1.-	Lipopolysaccharide
FTT0436c	lpxH	3.6.1.-	Lipopolysaccharide
FTT0450	mraY	2.7.8.13	Peptidoglycan
FTT0697	ftsI	2.4.1.129	Peptidoglycan biosynthesis
FTT0701	kdsA	2.5.1.55	Lipopolysaccharide
FTT0811c	murG	2.4.1.227	Peptidoglycan
FTT1027c	yrbI	3.1.3.45	Lipopolysaccharide
FTT1478c	kdsB	2.7.7.38	Lipopolysaccharide
FTT1561	kdtA	2.-.-.-	Lipopolysaccharide
FTT1568c	lpxB	2.4.1.182	Lipopolysaccharide
FTT1569c	lpxA	2.3.1.129	Lipopolysaccharide
FTT1571c	lpxD	2.3.1.-	Lipopolysaccharide
FTT1681c	lpcA	5.-.-.-	Lipopolysaccharide
FTT0318	cdsA	2.7.7.41	Glycerophospholipid
FTT0320	pgsA	2.7.8.5	Glycerophospholipid
FTT0384c	psd	4.1.1.65	Glycerophospholipid
FTT0782	fabI	1.3.1.9	Fatty acid biosynthesis
FTT0871	gpsA	1.1.1.94	Glycerophospholipid
FTT1374		2.3.1.39	Fatty acid biosynthesis
FTT1375	fabG	1.1.1.100	Fatty acid biosynthesis, Biosynthesis of unsaturated fatty acids
FTT1377	fabF	2.3.1.41	Fatty acid biosynthesis
FTT1570c	fabZ	4.2.1.-	Fatty acid
FTT0437c	pyrE	2.4.2.10	Pyrimidine
FTT0532c	nrdB	1.20.4.1	Purine, Pyrimidine
FTT0534c	nrdA	1.17.4.1	Purine, Pyrimidine
FTT0559c	cmk	2.7.4.14	Pyrimidine
FTT0893	purM	6.3.3.1	Purine
FTT0894	purCD	6.3.4.13	Purine
FTT0909		6.3.5.2	Purine
FTT1019c	guaA	6.3.4.1	Purine
FTT1161	adk	2.7.4.3	Purine
FTT1317c	guaB	1.1.1.205	Purine
FTT1470c	gmk	2.7.4.8	Purine
FTT1648c	pyrF	4.1.1.23	Pyrimidin
FTT1720c	purL	6.3.5.3	Purine
FTT0897	purK	4.1.1.21	Purine
FTT0374c	pyrG	6.3.4.2	Pyrimidine
FTT0373c	ndk	2.7.4.6	Purine, Pyrimidine
FTT1197c	murI	5.1.1.3	D-Glutamine and D-glutamate
FTT0317	uppS	2.5.1.31	Terpenoid backbone biosynthesis
FTT1456c	wbtH	6.3.5.4	Alanine, aspartate and glutamate, Nitrogen
FTT0420	murE	6.3.2.13	Lysine, Peptidoglycan biosynthesis
FTT0422	murF		Lysine, Peptidoglycan biosynthesis
FTT0431	speE	2.5.1.16	Cysteine and methionine, Arginine and proline, beta-Alanine, Glutathione
FTT0388	glmS	2.6.1.16	Alanine, aspartate and glutamate, Amino sugar and nucleotide sugar
FTT0372c	accD	6.4.1.2	Fatty acid biosynthesis, Pyruvate, Propanoate, Carbon fixation
FTT0473	accC	6.4.1.2	Fatty acid biosynthesis, Pyruvate, Propanoate, Carbon fixation
FTT1498c	accA	6.4.1.2	Fatty acid, Pyruvate, Propanoate, Carbon fixation

Locus Name	Gene Symbol	EC Number	Pathway
FTT1304c	murB	1.1.1.158	Amino sugar and nucleotide sugar, Peptidoglycan biosynthesis
FTT1305c	murA	2.5.1.7	Amino sugar and nucleotide sugar, Peptidoglycan biosynthesis
FTT0472	accB	6.4.1.2	Fatty acid biosynthesis, Pyruvate, Propanoate
FTT0674	prsA	2.7.6.1	Pentose phosphate, Purine
FTT0674	prsA	2.7.6.1	Pentose phosphate, Purine
FTT1249	nadE	6.3.5.1	Nicotinate and nicotinamide, Nitrogen
FTT1721c	purF	2.4.2.14	Purine, Alanine, aspartate and glutamate
FTT0766	deoD	2.4.2.1	Purine, Pyrimidine, Nicotinate and nicotinamide
FTT0489c	trxB	1.8.1.9	Pyrimidine, Selenocompound
FTT1390	panC	6.3.2.1	beta-Alanine, Pantothenate and CoA biosynthesis
FTT1391	panD	4.1.1.11	beta-Alanine, Pantothenate and CoA biosynthesis
FTT0451	murD	6.3.2.9	D-Glutamine and D-glutamate, Peptidoglycan
FTT0015	purB	4.3.2.2	Purine, Alanine, aspartate and glutamate
FTT0113	deoB	5.4.2.7	Pentose phosphate
FTT0117	tmk	2.7.4.9	Pyrimidine
FTT0203c	purH	3.5.4.10	Purine
FTT0219c			Transport
FTT0238	aroE1	1.1.1.25	Phenylalanine, tyrosine and tryptophan
FTT0239	murC	6.3.2.8	D-Glutamine and D-glutamate, Peptidoglycan
FTT0251	ilvE	2.6.1.42	Valine, leucine and isoleucine
FTT0286c	lpxD2	2.3.1.-	Lipopolysaccharide
FTT0415	glgC		
FTT0426	thrA		
FTT0533c	grxA		
FTT0643	ilvC		
FTT0650c	grxB		
FTT0767c			
FTT0788c	kdsD	5.3.1.13	
FTT1124	metIQ		
FTT1174c			
FTT1688			
