# Supplementary material for: Rapid Countermeasure Discovery against Francisella tularensis Based on a Metabolic Network Reconstruction
Source: PLoS One. 2013 May 21;8(5):e63369. doi: 10.1371/journal.pone.0063369 (PMC3660459; doi:10.1371/journal.pone.0063369)
Supplement: Table S2 — PBPK results. Column 1 shows the ChemNavigator structure identifier, column 2 shows the molecular SMILES code, column 3 shows a picture of the molecule, column 4 shows the volume of distribution (Vd; in l/kg), column 5 shows the clearance (CL; in ml·min−1·kg−1), column 6 shows the mean residence time (MRT; in h), column 7 shows the half-life (T ½; in h), and column 8 shows the putative targets(s). (PDF) [file pone.0063369.s003.pdf]

Structure_ID	SMILES	Structure	VD	CL	MRT	T1/2	Target
27928881	SC1=NC(N)=NC2=C1NC=N2	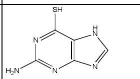	0.61	1.24	8.22	5.69	aroC
28433997	SC1=NC2=C(O)N=C(N)N=C2N1	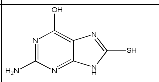	0.56	1.44	6.44	4.46	aroC
29515579	NC1=NC(N([C@@H]2C[C@H](CO)C=C2)C=N3)=C3C(NC4CC4)=N1	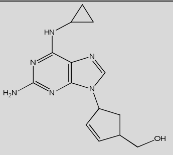	0.75	1.38	9.04	6.26	aroC
28763139	ON(C(N)=O)C(C)C1=CC2=CC=CC=C2S1	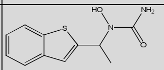	2.67	3.16	14.07	9.75	aroC
27144340	O=C1C2=C(S/C1=C\C3=CC=C(O)C=C3)C=CC=C2	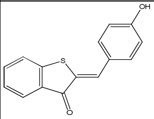	3.28	3.71	14.75	10.22	aroC
30013689	OC1=CC(O/C2=C\C3=CC(O)=C(O)C(O)=C3)=C(C2=O)C(O)=C1	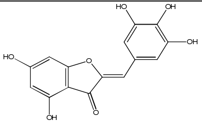	2.09	2.81	12.41	8.60	aroC
30979018	O=C(COC1=CC(O/C2=C\C3=CC=CC=C3)=C(C2=O)C=C1)O	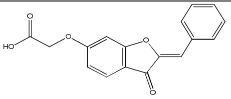	0.36	2.59	2.35	1.63	aroC
34736825	OC1=CC(O/C2=C/C3=CC=CN=C3)=C(C2=O)C=C1	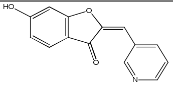	1.82	2.73	11.13	7.71	aroC
34775739	OC1=CC(O/C2=C\C3=CC=C(C(C)(C)C)C=C3)=C(C2=O)C=C1	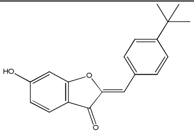	4.90	4.00	20.44	14.16	aroC
38986575	OC1=CC(O/C2=C\C3=CNC4=C3C=CC=C4)=C(C2=O)C=C1	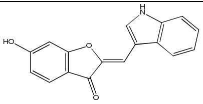	3.25	3.20	16.93	11.73	aroC
87524429	CCC1=CC(C2=O)=C(O/C2=C\C3=CC(O)=CC=C3)C=C1	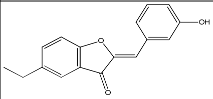	3.35	4.01	13.93	9.66	aroC
107441709	OC1=CC=C2C(O/C(C2=O)=C/C3=CC=C(C=C3)N4C=NC=C4)=C1	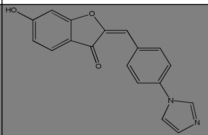	1.17	1.96	9.94	6.89	aroC
126767814	FC1=C(N2C=NC=C2)C=CC(/C=C(C3=O)/OC4=C3C=CC(O)=C4)=C1	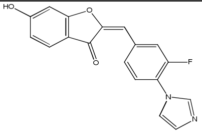	1.26	2.09	10.01	6.93	aroC
131479406	[O-]C(CC/C(C)=C/CC1=C(O)C2=C(COC2=O)C(C)=C1OC)=O	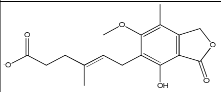	0.44	2.50	2.93	2.03	aroC
28850331	FC(F)(C1=NN(C2=CC=C(S(=O)(N)=O)C=C2)C(C3=CC=C(C)C=C3)=C1)F	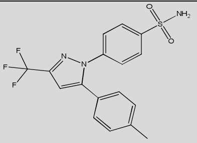	2.79	3.00	15.49	10.73	aroC

29512152	OC1=C(C2=NC(C3=C(O)C=CC=C3)=NN2C4=CC=C(C(O)=O)C=C4)C=CC=C1	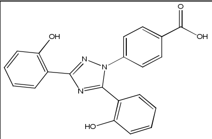	0.84	2.57	5.45	3.78	aroC
180903688	O=P([O-])([O-])OCN(C1=O)C(NC1(C2=CC=CC=C2)C3=CC=CC=C3)=O	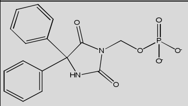	0.28	2.41	1.96	1.36	aroC
249758050	NC(C(O)=O)(CC1=CC=CS1)C	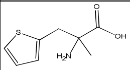	0.47	0.99	7.93	5.50	aroG
27644889	N[C@H](CC1=CC=CS1)C(O)=O	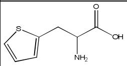	0.48	0.87	9.10	6.31	aroG
29254073	CC(S1)=CC=C1CC(N)C(O)=O	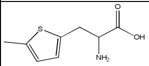	0.54	0.67	13.50	9.35	aroG
29254075	NC(C(O)=O)CC1=CC=C(Br)S1	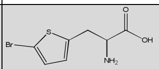	0.58	0.65	14.86	10.30	aroG
29254076	CC1=C(SC=C1)CC(N)C(O)=O	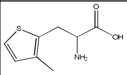	0.51	0.81	10.46	7.25	aroG
152416852	NC(CC(S1)=CC=C1Cl)C(O)=O	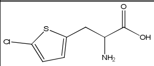	0.55	0.68	13.57	9.40	aroG
161743282	BrC1=CSC(CC(N)C(O)=O)=C1	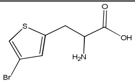	0.55	0.71	12.88	8.93	aroG
30379056	NC(CC1=CC=CO1)C(O)=O	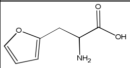	0.47	0.96	8.06	5.59	aroG
249754974	NC(C(O)=O)(CC1=CC=CO1)C	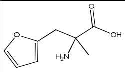	0.45	1.04	7.27	5.04	aroG
129062300	NC(CC(O1)=CC=C1C)C(O)=O	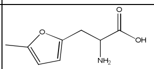	0.50	0.72	11.70	8.10	aroG
187215823	NC(CC1=CC=NO1)C(O)=O	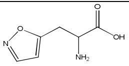	0.46	1.05	7.39	5.12	aroG
39432154	NC(C(O)=O)CC1=CC(C)=NO1	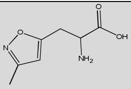	0.49	0.85	9.63	6.67	aroG
28433075	N[C@H](CC1=CSC=C1)C(O)=O	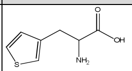	0.46	0.98	7.77	5.38	aroG
29292508	NC(CC1=CSC=C1)C(O)=O	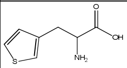	0.46	0.98	7.77	5.38	aroG
161768273	NC(CC1=CC=CN1C)C(O)=O	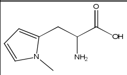	0.45	1.05	7.19	4.98	aroG
30380340	OC1C=CC(C(O)=O)(C=C1)CC(C(O)=O)=O	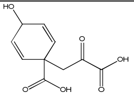	0.14	1.27	1.80	1.25	aroG
28809758	OC(C1=C[C@@H](OC(C(O)=O)=C)[C@H](O)C=C1)=O	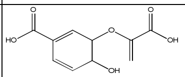	0.14	1.01	2.38	1.65	aroG
61889507	N[C@H]1[C@H](OC(C(O)=O)=C)C=C(C=C1)C(O)=O	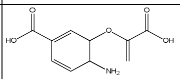	0.15	1.02	2.42	1.67	aroG

29142037	O=CC$_1$=CC=C(C(OC)=C$_1$)OCC$_2$=CC=CC=C$_2$COC$_3$=CC=C(C=C$_3$OC)/C=C(C(N$_4$)=O)/SC$_4$=S	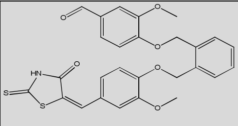	3.91	3.15	20.67	14.32	aroK
29146142	COC$_1$=CC=C(CCNC(NCC$_2$=CC(CNC(NCCC$_3$=CC=C(OC)C=C$_3$)=O)=CC=C$_2$)=O)C=C$_1$	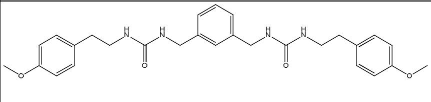	2.36	2.78	14.16	9.81	aroK
29300286	ClC$_1$=CC$_2$=NC=NC(OC$_3$=NC=C(NC(NC$_4$=CC(OC$_5$CCCC$_5$)=C(OC)C=C$_4$)=O)C=C$_3$)=C$_2$C=C$_1$	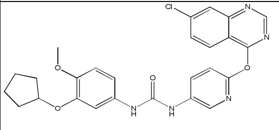	3.23	3.67	14.66	10.16	aroK
29720610	O=[N+]([O-])C$_1$=CC(/C=C/C(NC$_2$=CC(C(O$_3$)=NC$_4$=C$_3$C(C)=CC(C)=C$_4$)=CC=C$_2$)=O)=CC=C$_1$	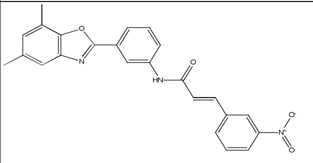	2.74	2.96	15.44	10.70	aroK
29864729	O=[N+](C$_1$=CC=C(/C=C(C$_2$=O)\SC(NC$_3$=CC=CC$_4$=CC=CC=C$_{34}$)=C$_2$C(OCC)=O)C=C$_1$)[O-]	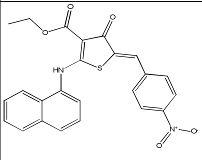	2.89	3.09	15.61	10.82	aroK
30224149	O=C(NC$_1$=CC=C(NC(NC$_2$=CC=CC=C$_2$)=O)C=C$_1$)C$_3$=CC=C(NC(NC$_4$=CC=CC=C$_4$)=O)C=C$_3$	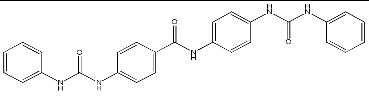	3.14	3.43	15.26	10.58	aroK
30233573	O=[N+]([O-])C$_1$=CC(/C=C/C(NC$_2$=CC(C(O$_3$)=NC$_4$=C$_3$C=CC(CC)=C$_4$)=CC=C$_2$)=O)=CC=C$_1$	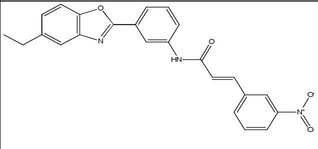	3.00	3.20	15.61	10.82	aroK
30169904	ClC$_1$=CC(C(O)=O)=C(C(O$_2$)=CC=C$_2$/C=C$_3$C(N(NC$\backslash_3$=O)C$_4$=CC=CC=C$_4$)=O)C=C$_1$	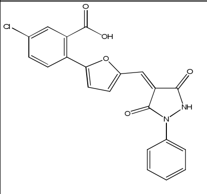	0.12	2.80	0.71	0.50	aroK
31629252	FC$_1$=CC=C(N(C/$_2$=O)NC(C$_2$=C\C$_3$=CC=C(C$_4$=CC=C(C(N)=O)C=C$_4$)O$_3$)=O)C=C$_1$	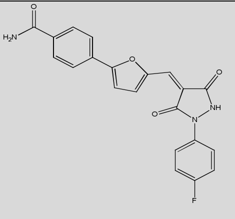	0.66	2.44	4.51	3.12	aroK
30240165	ClC$_1$=CC(N(NC$_2$=O)C(/C$_2$=C\C(O$_3$)=CC=C$_3$C$_4$=C(C(O)=O)C=CC=C$_4$)=O)=CC=C$_1$	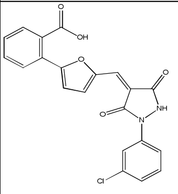	0.12	2.80	0.72	0.50	aroK
30240865	BrC$_1$=CC=CC(N(C/$_2$=O)NC(C$_2$=C\C$_3$=CC=C(C$_4$=CC=CC=C$_4$C(O)=O)O$_3$)=O)=C$_1$	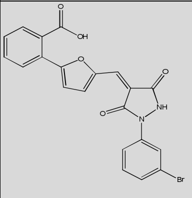	0.12	2.80	0.71	0.49	aroK

30272448	BrC1=CC(C(O)=O)=C(C(O2)=CC=C2/C=C3C(N(NC\3=O)C4=CC(C)=CC=C4)=O)C=C1	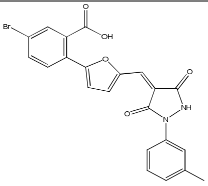	0.11	2.81	0.66	0.46	aroK
35357007	BrC1=CC(C(O)=O)=C(C(O2)=CC=C2/C=C3C(N(NC\3=O)C4=CC=C(CC)C=C4)=O)C=C1	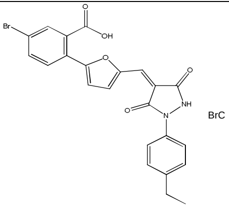	0.16	2.81	0.94	0.65	aroK
39446728	BrC1=CC(C(O)=O)=C(C(O2)=CC=C2/C=C3C(N(NC\3=C)C4=CC=C(C)C=C4)=O)C=C1	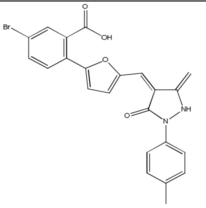	0.29	2.79	1.75	1.21	aroK
39446743	BrC1=CC(C(O)=O)=C(C(O2)=CC=C2/C=C3C(N(NC\3=C)C4=CC(C)=C(C)C=C4)=O)C=C1	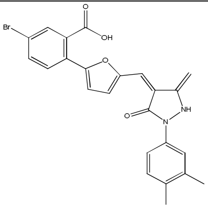	0.37	2.78	2.22	1.54	aroK
75601322	FC1=CC=C(N(NC2=O)C(/C2=C\C(O3)=CC=C3C4=C(C([O-])=O)C=CC=C4)=O)C=C1	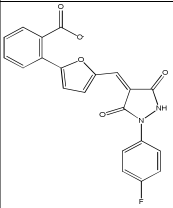	0.13	2.78	0.80	0.56	aroK
76152425	ClC1=CC(N(NC2=O)C(/C2=C\C(O3)=CC=C3C4=C(C([O-])=O)C=CC=C4)=O)=CC=C1	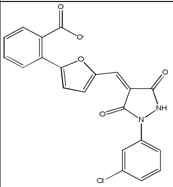	0.12	2.80	0.72	0.50	aroK
132064348	ClC1=CC(C([O-])=O)=C(C(O2)=CC=C2/C=C3C(N(NC\3=O)C4=CC=CC=C4)=O)C=C1	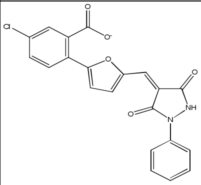	0.12	2.80	0.71	0.49	aroK
28877955	BrC1=CC(C(O)=O)=C(C(O2)=CC=C2/C=C3C(N(NC\3=O)C4=CC=C(C(OCC)=O)C=C4)=O)C=C1	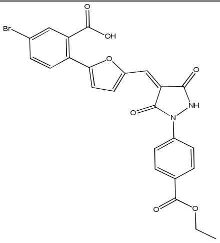	0.12	2.80	0.69	0.48	aroK

29152838	BrC1=CC(C(O)=O)=C(C(O2)=CC=C2/C=C3C(N(NC\3=O)C4=CC(Cl)=C(Cl)C=C4)=O)C=C1	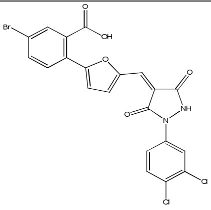	0.10	2.81	0.61	0.42	aroK
28267724	ClC1=CC=C(/C=C(OC2=C(O)C=CC=C2)/C(C3=CC=CC=C3)=O)C=C1	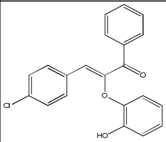	2.69	2.90	15.43	10.69	aroK
30188195	O=C1N2N=CC=C2NC3=C1C=CC=C3	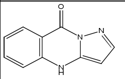	3.14	4.07	12.88	8.93	coaD
70542065	O=C1N2N=C(C=C2NC3=C1C=CC=C3)O	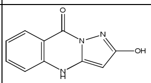	3.25	4.14	13.08	9.06	coaD
102132334	O=C1N2NC(C=O)=CC2=NC3=C1C=CC=C3	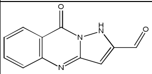	0.86	1.09	13.11	9.09	coaD
27639774	O=C1N2N=CC(C=O)=C2NC3=CC=CC=C31	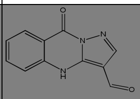	2.76	3.28	14.06	9.74	coaD
238712227	O=C1N2N=C(C)C(C=O)=C2NC3=CC=CC=C31	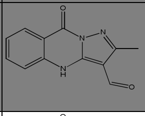	2.59	2.94	14.70	10.19	coaD
238713396	O=C1N2N=C(C(CN)=C2NC3=C1C=CC=C3)C	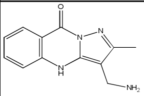	0.73	2.44	5.02	3.48	coaD
147911620	O=C1N2N=CC(/C=N/C3=CC=C(O)C=C3)=C2NC4=C1C=CC=C4	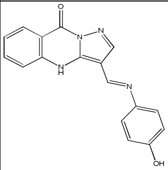	3.38	4.01	14.06	9.74	coaD
148067159	O=C1N2N=CC(/C=N/N)=C2NC3=C1C=CC=C3	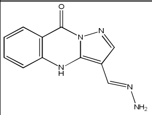	2.12	2.61	13.55	9.39	coaD
148227647	O=C1N2N=CC(CN=O)=C2NC3=C1C=CC=C3	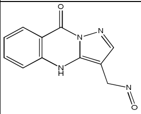	2.88	3.94	12.19	8.45	coaD
148936594	O=C1N2N=C(C=C2NC3=C1C=CC=C3)C(OCC)=O	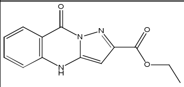	3.14	3.54	14.77	10.24	coaD
187236494	COC1=CC=CC2=C1NC3=CC(C(N)=O)=NN3C2=O	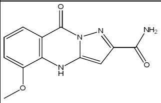	1.86	2.59	11.97	8.30	coaD
187236496	COC1=CC=CC2=C1NC3=CC(C#N)=NN3C2=O	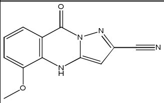	3.05	3.66	13.86	9.61	coaD

187236497	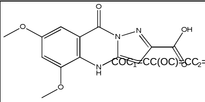 COC1=CC(OC)=CC2=C1NC3=CC(C(O)=O)=NN3C2=O	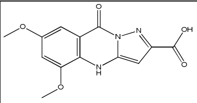	0.30	2.43	2.06	1.43	coaD
187236498	OC1=CC=CC2=C1NC3=CC(C(O)=O)=NN3C2=O	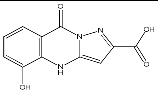	0.29	2.42	2.03	1.40	coaD
295773423	CC1=CC2=CC=CC=C2N1CC(NCC3=CC=CN=C3N(C)C)=O	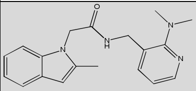	2.50	3.05	13.65	9.46	fabI
29504564	O=C(C1=CC=CC=C12)N(CC3=CC=CC=C3N)C2=O	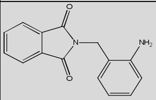	2.51	3.19	13.10	9.08	fabI
35777990	FC1=CC=C(NC(CN2C3=CC=CC=C3C(/C=C/C4=C([N+]([O-])=O)C(O)=NC(O)=N4)=C2)=O)C=C1	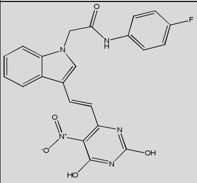	3.11	3.34	15.48	10.73	fabI
35957082	O=S(NC1=CC(Cl)=C(Cl)C=C1)(C2=CC=C(NC(NC3=CC=C([N+]([O-])=O)C=C3)=O)C=C2)=O	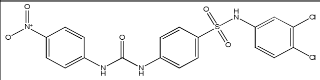	2.99	2.93	16.97	11.76	fabI
38362581	O=S(NC1=CC=CC=C1)(C2=CC=C(NC(NC3=CC=C([N+]([O-])=O)C=C3)=O)C=C2)=O	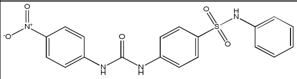	2.63	2.86	15.34	10.63	fabI
236546277	FC1=CC(C#N)=CC=C1OC2=CC=CC=C2O	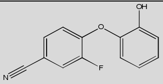	3.51	4.31	13.56	9.40	fabI
236546278	FC1=CC(C(N)=O)=CC=C1OC2=CC=CC=C2O	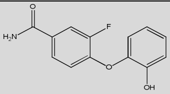	2.88	3.51	13.67	9.47	fabI
28811136	NC(N=C(C1=N2)O)=NC1=NC=C2CO	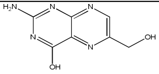	0.48	1.01	7.86	5.45	folK
27928841	OC1=C(C(N=C2)=NC(N)=N1)N=C2CNC3=CC=C(C(O)=O)C=C3	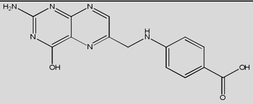	0.37	1.47	4.18	2.90	folK
30385311	O=C(C1=CC=C(NCCC2=CNC3=NC(N)=NC(O)=C3N2)C=C1)O	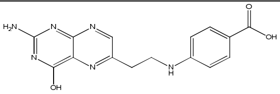	0.44	0.81	9.10	6.31	folK
30408296	NC(N=C(C1=N2)O)=NC1=NC(O)=C2C(O)=O	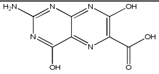	0.40	1.68	3.99	2.77	folK
68377661	O=P(O)(OCC1=NC2=C(N=C(N=C2O)N)N=C1)O	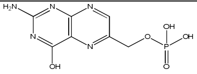	0.21	1.01	3.52	2.44	folK
77912390	NC(C1=N2)=NC(N)=NC1=NC=C2CNC3=CC=C(C([O-])=O)C=C3	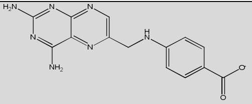	0.44	0.81	9.07	6.29	folK

70078879	O=C1NC(N)=NC2NC=C(NC12)C(C(O)C)=O	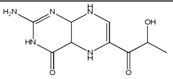	0.45	1.10	6.90	4.78	folK
30445429	O=C(C1=CN(C2=NC(N)=NC(O)=C2N1)C)O	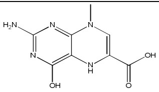	0.44	1.13	6.47	4.49	folK
313845810	O[C@H](C1=CNC2=C(O)N=C(N)N=C2N1)[C@@H](CO)O	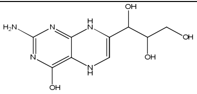	0.45	1.10	6.81	4.72	folK
187140031	C[C@@H]1NC2=C(NC1)N=C(N)N=C2O	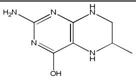	0.64	1.27	8.36	5.79	folK
135968811	OC1=NC(N)=NC2=C1NC(CN2)C3CC3	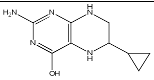	0.85	1.58	8.95	6.20	folK
28775227	OC1=NC(N)=NC2=C1NC(C(N2)C)C	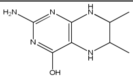	0.86	1.60	8.95	6.20	folK
68371377	OC1=NC(N)=NC2=C1NC([C@@](C)(N2)CCC3=CC=CC=C3)C=O	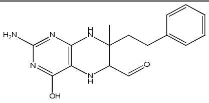	2.42	3.12	12.94	8.97	folK
129802050	CC1=CC=C(C=C1)NC[C@@H]2NC3=C(NC2C)N=C(N)N=C3O	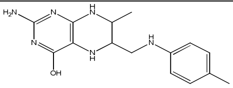	2.37	3.13	12.61	8.74	folK
129825112	OC1=NC(N)=NC2=C1NC(CN2)C([C@H](C)O)=O	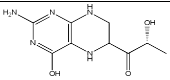	0.48	1.06	7.54	5.22	folK
28918893	C/C(C(O)=O)=C(C1=O)/NN(C2=C1C(O)=NC(N)=N2)C	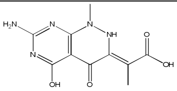	0.36	0.55	10.94	7.58	folK
313844357	OC1=NC(N)=NC2=C1N(C(CNC3=CC=C(C(N[C@@H](C(O)=O)CCC(O)=O)=O)C=C3)=CN2)CO	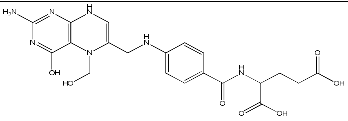	0.15	0.56	4.53	3.14	folK
73521837	O=P(COCCN(C(N)=NC1=NC=CN=C21)C2=O)(O)O	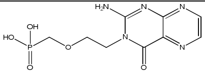	0.17	1.04	2.75	1.90	folK
136746903	O=C(CC/C=C(C1=O)\NC2=C(O)N=C(O)N=C2N1C[C@@H]([C@@H]([C@@H](CO)O)O)O)O	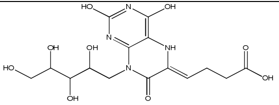	0.15	0.99	2.44	1.69	folK
29508912	O=C(CC/C=C(C1=O)\NC2=C(O)N=C(O)N=C2N1C[C@@H]([C@@H]([C@@H](CO)O)O)O)O	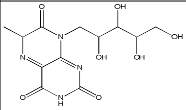	0.24	1.10	3.59	2.49	folK
27678823	O=C(N)C1=C(C(N)=O)N=CN1	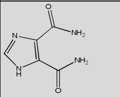	0.41	1.03	6.60	4.57	folK
30380846	NC(CC1=CSC=N1)C(O)=O	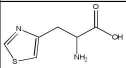	0.46	1.02	7.48	5.18	folK

130533128	O=P(O)(OCC(C([C@@H](CN$_1$C$_2$=C(N=C$_3$C$_1$=NC(NC$_3$=O)=O)C=C(C)C(C)=C$_2$)O)O)O)O	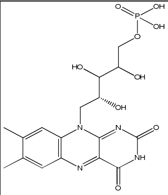	0.15	0.52	4.96	3.44	folK
131690280	O=P(OCC(C([C@@H](CN$_1$C$_2$=C(N=C$_3$C$_1$=NC(NC$_3$=O)=O)C=C(C)C(C)=C$_2$)O)O)O)([O-])[O-]	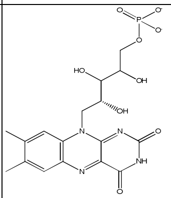	0.15	0.51	5.08	3.52	folK
131690283	O=P(OCC(C([C@@H](CN$_1$C$_2$=C(N=C$_3$C$_1$=NC(NC$_3$=O)=O)C=C(C)C(C)=C$_2$)O)O)O)([O-])[O-]	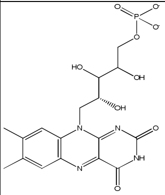	0.15	0.51	5.08	3.52	folK
153990598	NC(C$_1$O)C(OC(C$_1$O)CO)O	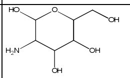	0.42	1.16	5.97	4.14	lpcA
101352797	O[C@@H](C$_1$O)OC([C@H](C$_1$O)O)CO	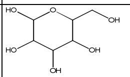	0.45	1.13	6.68	4.63	lpcA
158582040	N[C@@H](C$_1$O)OC[C@@H](O)C$_1$O	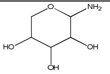	0.52	1.14	7.56	5.24	lpcA
34733326	ClC$_1$=C(OC(C$_2$O)OC(C(C$_2$O)O)CO)C=CC(Cl)=C$_1$	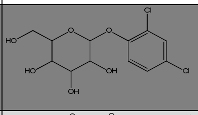	0.90	1.41	10.68	7.40	lpcA
77912390	OCC$_1$OC(OC$_2$=CC=C(C)C(C)=C$_2$)C(O)C(O)C$_1$O	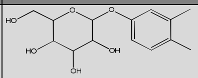	0.80	1.30	10.25	7.10	lpcA
27654666	OC$_1$C(OC(CO)C(O)C$_1$O)NC$_2$=CC=CC=C$_2$	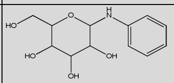	0.49	0.89	9.13	6.32	lpcA
27119828	OC(C$_1$O)C(NC$_2$=CC(O)=CC=C$_2$)OC(C$_1$O)CO	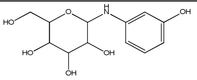	0.47	0.93	8.51	5.89	lpcA
117776378	BrC$_1$=C(C)C=C(NC(C$_2$O)OC(C(C$_2$O)O)CO)C=C$_1$	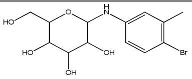	0.75	1.08	11.66	8.08	lpcA
27654450	OC$_1$C(OC(CO)C(O)C$_1$O)NC$_2$=CC=C(OCC)C=C$_2$	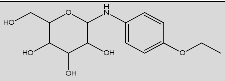	0.50	0.92	9.04	6.26	lpcA
27657439	OC(C$_1$O)C(NC$_2$=CC(C)=C(C)C=C$_2$)OC(C$_1$O)CO	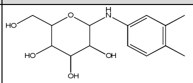	0.65	0.96	11.31	7.84	lpcA
27928841	O=[N+](C$_1$=CC=C(NC$_2$OC(CO)C(O)C(O)C$_2$O)C=C$_1$)[O-]	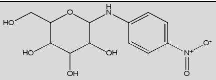	0.50	0.82	10.20	7.07	lpcA
28116592	BrC$_1$=CC=C(N[C@@H]([C@@H]$_2$O)O[C@H](CO)[C@@H](O)[C@@H]$_2$O)C=C$_1$	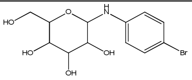	0.59	0.84	11.76	8.15	lpcA

30424932	OC(C1O)C(NC2=CC=C(OCC)C=C2)OC(C1O)CO	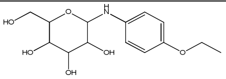	0.50	0.92	9.04	6.26	lpcA
34694328	OC(C1O)C(NC2=CC=C(C(C)=O)C=C2)OC(C1O)CO	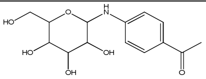	0.53	0.60	14.76	10.23	lpcA
60172860	OC(C1O)C(NC2=CC(OC)=CC=C2)OC(C1O)CO	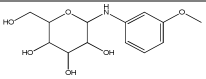	0.49	0.88	9.18	6.36	lpcA
28803460	O[C@H]1[C@H](O)[C@@H](O)[C@@H](OC1=O)CO	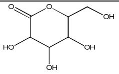	0.45	1.11	6.72	4.66	lpcA
30012668	O[C@@H]([C@@H]1O)O[C@@H]([C@@H]([C@@H]1O)O)CO	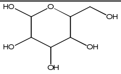	0.45	1.13	6.68	4.63	lpcA
27592880	OC1C(OCC(O)C1O)NC2=CC=C(C)C=C2	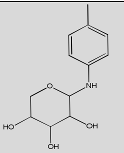	0.69	1.15	9.98	6.92	lpcA
34733326	BrC1=CC=C(N [illegible] 2OC [illegible] (O) [illegible] (O) [illegible] 2O)C=C1	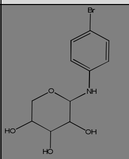	0.80	1.28	10.43	7.23	lpcA
28764874	O=P(O)(O[C@H]1[C@@H](O)[C@H](O)CC(C(O)=O)=C1)O	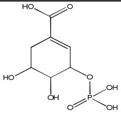	0.15	0.95	2.57	1.78	lpcA
28780304	O=P(O)(O[C@H]1[C@@H](O)[C@H](OC(C(O)=O)=C)CC(C(O)=O)=C1)O	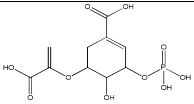	0.15	0.59	4.36	3.02	lpcA
70787116	F[C@H]1[C@@H](OC(C(O)=O)=C)[C@H](O)[C@H](OP(O)(O)=O)C=C1C(O)=O	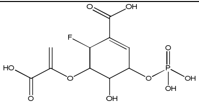	0.15	0.60	4.25	2.94	lpcA
30424932	OC1=C(C(N)=O)N=CN1[C@@H]2O[C@H](CO)[C@@H](O)[C@H]2O	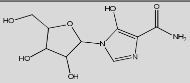	0.43	1.13	6.43	4.45	lpcA
27593512	NC1=C(C(N)=O)N=CN1[C@@H]2O[C@H](CO)C(O)C2O	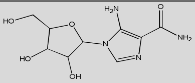	0.44	1.14	6.49	4.50	lpcA
27737380	O=[N+]([O-])C1=NC=CC=C1O	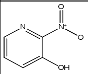	0.44	2.00	3.66	2.54	nadC
28665953	O=C(C1=CC=CN=C1)[O-]	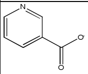	0.41	1.02	6.63	4.59	nadC
28667598	OC1=CC=CN=C1C(OC)=O	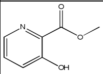	2.00	2.82	11.85	8.21	nadC
30019459	NC(COC1=CC=CN=C1C(O)=O)=O	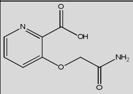	0.42	0.84	8.32	5.77	nadC

???

70358905	O=[N+]([O-])C$_1$=NC=CC=C$_1$C(N)=O	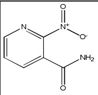	0.72	1.30	9.19	6.37	nadC
234796908	O=[N+]([O-])C$_1$=NC=CC=C$_1$C=O	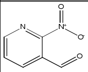	1.56	2.29	11.33	7.85	nadC
30409034	O=[N+]([O-])C$_1$=NC=CC=C$_1$C(O)=O	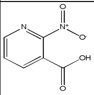	0.32	1.87	2.87	1.99	nadC
78519792	NC$_1$=NC=CC=C$_1$C([O-])=O	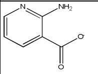	0.41	0.84	8.17	5.66	nadC
70624296	SC$_1$=C(C(O)=O)N=CC=C$_1$	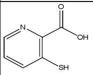	0.46	0.77	9.85	6.83	nadC
131501422	OC$_1$=CC=CN=C$_1$C([O-])=O	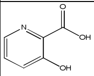	0.45	0.63	11.89	8.24	nadC
247608578	NC(COC$_1$=C(C(O)=O)N=CC=C$_1$)=O	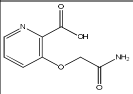	0.42	0.84	8.32	5.77	nadC
247608587	O=C(C$_1$=C(OCCN(C)C)C=CC=N$_1$)O	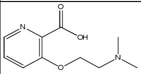	0.45	1.08	6.95	4.81	nadC
247609672	OCCCOC$_1$=CC=CN=C$_1$C(O)=O	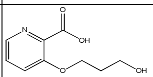	0.45	0.74	10.18	7.06	nadC
161604885	O=C(C$_1$=CC=CN=C$_1$C(OC(C)C)=O)O	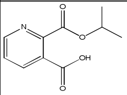	0.31	2.15	2.40	1.66	nadC
92156924	O=S(N)(C$_1$=C(C(OC)=O)N=CC=C$_1$)=O	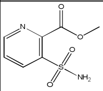	0.58	0.62	15.57	10.79	nadC
30466351	O=C(C$_1$=NC(O)=CC=C$_1$C(O)=O)O	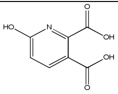	0.22	1.63	2.20	1.52	nadC
183986600	NC$_1$=NC(N)=C(CC$_2$=CC(OC)=C(OC)C(OC)=C$_2$)C=N$_1$	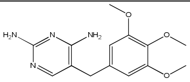	1.21	2.50	8.03	5.56	nadC
29515878	O=C(OCC)C$_1$=C(C(OCC)=O)C=C(C(CCC$_2$)=O)C$_2$=N$_1$	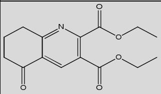	2.12	2.66	13.28	9.20	nadC
29716752	O=[N+]([O-])C$_1$=C(/C=C(C(C$_2$=CC=CC=C$_2$)=O)\C#N)C=CC=C$_1$	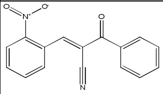	2.45	2.78	14.66	10.16	nadC
68372779	ClC$_1$=C(C$_2$=C(C(OC(C)C)=O)C(C)=[N+](CC)C(C(O)=O)=C$_2$C(O)=O)C=CC=C$_1$	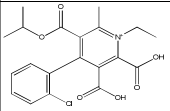	0.18	0.14	21.32	14.78	nadC
154011954	O=[N+]([O-])C$_1$=NC=C(C$_2$=CC=C(C(O)=O)C=C$_2$)C=C$_1$	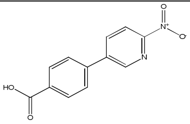	0.31	2.48	2.07	1.43	nadC

27328604	O=S(NC1=NC(C)=CC=N1)(C2=CC=C(NC(NC3=CC(Cl)=C(Cl)C=C3)=O)C=C2)=O	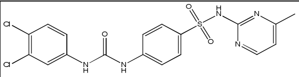	1.19	2.59	7.65	5.30	nadE
27531718	FC1=C(F)C(F)=C(C(O2)=NC3=C2C=CC(NC(C4=CC([N+]([O-])=O)=CC=C4)=O)=C3)C(F)=C1F	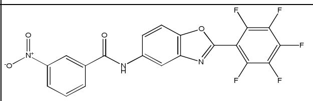	3.12	3.38	15.40	10.67	nadE
28436162	O=C1C[C@@H](C2=CC=C(OC)C(O)=C2)OC3=CC(O)=CC(O)=C13	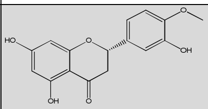	3.09	3.38	15.24	10.56	nadE
28759193	COC1=CC=CC(OC)=C1C(N[C@H]2[C@H](N3C2=O)SC(C)([C@@H]3C(O)=O)C)=O	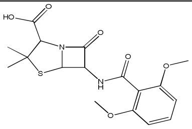	0.33	1.76	3.11	2.16	nadE
28759683	CC1([C@@H](N([C@@H]2S1)C([C@H]2NC(COC3=CC=CC=C3)=O)=O)C([O-])=O)C	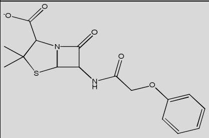	0.35	1.66	3.53	2.44	nadE
28765426	OC1=C(N=CN2COCCOC([C@H](C(C)C)N)=O)C2=NC(N)=N1	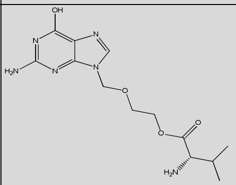	0.95	1.55	10.18	7.06	nadE
70610912	OC1=C(N=CN2COC(COC([C@H](C(C)C)N)=O)CO)C2=NC(N)=N1	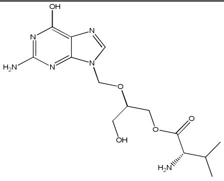	0.73	1.18	10.33	7.16	nadE
136822396	OC1=NC2=C(O)N=C(O)N=C2N1CC([C@@H]([C@@H](CO)O)O)O	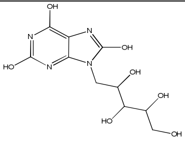	0.46	0.99	7.74	5.36	nadE
313845526	O=P(O)(OP(O)(O)=O)OC[C@@H]([C@@H]1O)O[C@@H](N2C3=NC=NC(N)=C3N=C2)[C@@H]1O	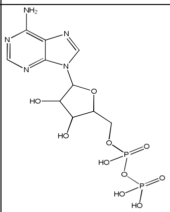	0.15	0.96	2.55	1.77	nadE
38201847	FC1=C(CSC2=NN=C3N2C(N)=NC(CCC)=C3)C(Cl)=CC=C1	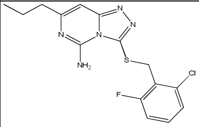	2.74	3.07	14.85	10.29	nadE
27560618	OC1=NC=NC2=C1C=NN2C(C3=CC4=C(C=CC=C4)C=C3O)=O	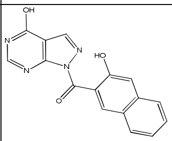	1.84	2.59	11.88	8.23	nadE

nadE or aroC???

105012384	FC1=CC=C(C(/C(C#N)=C/C2=C([N+]([O-])=O)C=CC=C2)=O)C=C1	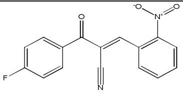	2.57	2.85	15.03	10.42	nadE
111735241	O=[N+](C1=CC=C(O)C(/C=N/C2=CC=CC(O)=C2)=C1)[O-]	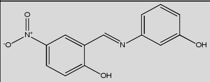	3.54	3.21	18.33	12.70	nadE
195983203	O=[illegible]([illegible])C1=CC(/C=N/C2=CC(O)=CC=C2)=C(O)C=C1	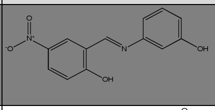	3.54	3.21	18.33	12.70	nadE
206592729	FC1=C(NC(NC2=CC=C(S(=O)(NC3=NC=CC=N3)=O)C=C2)=O)C=CC(C)=C1	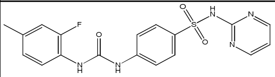	1.02	2.51	6.80	4.71	nadE
212465872	O=S(NC1=NC=CC=N1)(C2=CC=C(NC(NC3=C(C#N)C=CC(Cl)=C3)=O)C=C2)=O	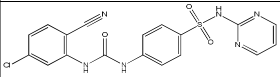	1.52	2.60	9.75	6.76	nadE
41513968	O=C(CSC1=NC(N2CCOCC2)=NC(NC3=CC=CC=C3)=N1)NC4=C(O)C=C([N+]([O-])=O)C=C4	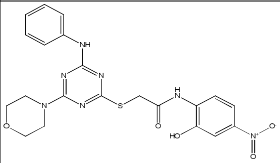	2.43	2.75	14.76	10.23	nadE
26883731	O=[N+]([O-])C1=CC=C(C(N2N=C(C=C2NC3=CC=CC=C3)C)=O)C=C1	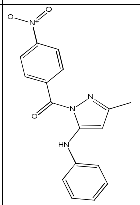	3.21	3.56	15.04	10.42	ribH
73966975	FC1=CC=C([C@]2(N(NC(C)=C2)C(C3=CC=CC=C3)=O)O)C=C1	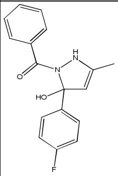	3.21	3.88	13.78	9.55	ribH
128161912	ClC1=CC=C([C@]2(N(NC(C)=C2)C(C3=C(C)C=CC=C3)=O)O)C=C1	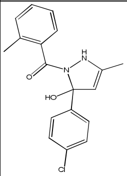	3.31	3.91	14.09	9.76	ribH
27388010	OC1(C2=CC=CC=C2)N(C(C3=CC=NC=C3)=O)NC(C)=C1	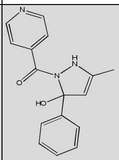	2.12	2.96	11.92	8.26	ribH
27556797	OC1(C2=CC=C(OC)C=C2)N(C(C3=CC=CC=C3)=O)NC(C)=C1	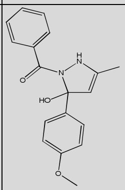	3.02	3.54	14.22	9.86	ribH

27832702	ClC1=CC=C(C(N2N=C(C)C=C2NC3=CC=CC=C3)=O)C=C1	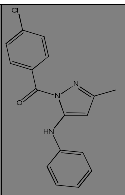	3.12	3.36	15.45	10.71	ribH
27907145	OC1(C2=CC=CC(OC)=C2)N(C(C3=CC=CC=C3)=O)NC(CC)=C1	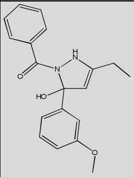	3.24	3.91	13.79	9.55	ribH
73921605	FC(C(N1)=C[C@@](C2=CC=CC=C2)(N1C(C3=CC=CC=C3)=O)O)(F)F	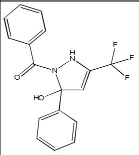	3.28	3.99	13.71	9.50	ribH
74224504	FC(C(N1)=C[C@@](C2=CC=C(F)C=C2)(N1C(C3=CC=CC=C3)=O)O)(F)F	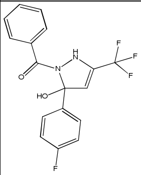	3.30	4.06	13.55	9.39	ribH
74224505	FC(C(N1)=C[C@@](C2=CC=C(F)C=C2)(N1C(C3=CC=C(C)C=C3)=O)O)(F)F	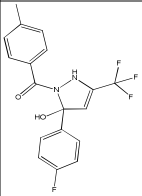	3.35	4.19	13.32	9.23	ribH
74235707	FC(C(N1)=C[C@@](C2=CC=C(Cl)C=C2)(N1C(C3=CC=NC=C3)=O)O)(F)F	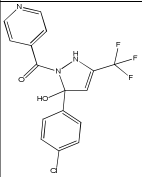	2.99	3.51	14.19	9.83	ribH
74269204	FC(C(N1)=C[C@@](C2=CC=C(F)C=C2)(N1C(C3=NC=CC=C3)=O)O)(F)F	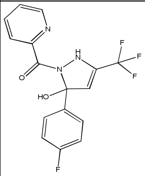	2.99	3.54	14.08	9.76	ribH
74357439	FC(C(N1)=C[C@@](C2=CC=C(Cl)C=C2)(N1C(C3=CN=CC=C3)=O)O)(F)F	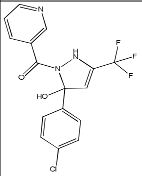	2.99	3.51	14.19	9.83	ribH
74387455	FC(C(N1)=C[C@@](C2=CC=C(F)C=C2)(N1C(C3=CC(OC)=CC=C3)=O)O)(F)F	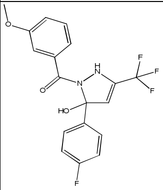	3.22	3.84	13.99	9.69	ribH

28568421	ClC1=CC=C(C(N2)=C[C@@](C)(N2C(C3=CC=C(Cl)C=C3)=O)O)C=C1	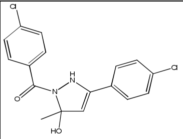	3.01	3.23	15.53	10.76	ribH
74035569	FC([C@]1(N(NC(C2=CC=CC=C2)=C1)C(C3=CC=CC=C3)=O)O)(F)F	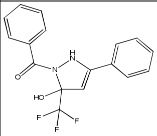	3.21	3.61	14.83	10.28	ribH
74449206	FC([C@]1(N(NC(C2=CC=C(Cl)C=C2)=C1)C(C3=CN=CC=C3)=O)O)(F)F	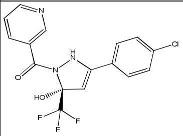	2.84	3.20	14.82	10.27	ribH
74742317	FC([C@]1(N(NC(C2=CC=C(Cl)C=C2)=C1)C(C3=CC(C)=CC=C3)=O)O)(F)F	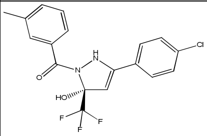	3.28	3.63	15.06	10.43	ribH
75585021	FC([C@]1(N(NC(C2=CC=C(F)C=C2)=C1)C(C3=CC(OC)=CC=C3)=O)O)(F)F	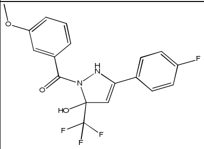	3.12	3.47	15.00	10.40	ribH
75588335	FC([C@]1(N(NC(C2=CC=C(F)C=C2)=C1)C(C3=CC(Cl)=CC=C3)=O)O)(F)F	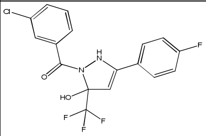	3.25	3.61	15.03	10.42	ribH
131983591	FC(C(N1)=C[C@@](C2=CC=C(F)C=C2)(N1C(C3=C(Cl)C=CC=C3)=O)O)(F)F	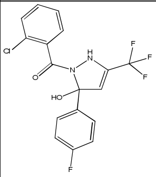	3.31	3.99	13.86	9.60	ribH
29401899	CC1=NN(C(NC2=CC=CC=C2)=C1)C(C3=CC=CC=C3)=O	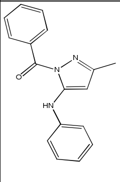	3.22	3.56	15.04	10.42	ribH
28246698	CC1=NN(C(NC2=CC=CC=C2)=C1)C(C3=CC=C(N)C=C3)=O	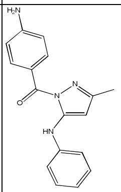	3.14	3.48	15.08	10.45	ribH
29365279	BrC1=CC=C(C(N2N=C(C=C2NC3=CC=CC=C3)C)=O)C=C1	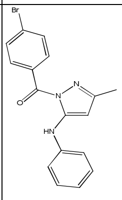	3.09	3.33	15.49	10.73	ribH

29401897	CC1=NN(C(NC2=CC=CC=C2)=C1)C(C3=CC=C(OC)C=C3)=O	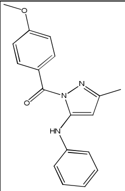	3.20	3.54	15.07	10.45	ribH
